# Bidirectional prefrontal-hippocampal dynamics organize information transfer during sleep in humans

**DOI:** 10.1038/s41467-019-11444-x

**Published:** 2019-08-08

**Authors:** Randolph F. Helfrich, Janna D. Lendner, Bryce A. Mander, Heriberto Guillen, Michelle Paff, Lilit Mnatsakanyan, Sumeet Vadera, Matthew P. Walker, Jack J. Lin, Robert T. Knight

**Affiliations:** 1 Helen Wills Neuroscience Institute, UC Berkeley, 132 Barker Hall, Berkeley, CA 94720 USA; 2 Dept. of Anesthesiology, University Medical Center Hamburg Eppendorf, Martinistrasse 52, 20246 Hamburg, Germany; 3 Dept. of Psychiatry and Human Behavior, UC Irvine, 101 The City Dr, Orange, CA 92868 USA; 4 Dept. of Neurology, UC Irvine, 101 The City Dr, Orange, CA 92868 USA; 5 Dept. of Neurosurgery, UC Irvine, 101 The City Dr, Orange, CA 92868 USA; 6 Dept. of Psychology, UC Berkeley, 2121 Berkeley Way, Berkeley, CA 94720 USA; 7 Dept. of Biomedical Engineering, Henry Samueli School of Engineering, 402 E Peltason Dr, Irvine, CA 92617 USA

**Keywords:** Non-REM sleep, Consolidation, Hippocampus, Slow-wave sleep

## Abstract

How are memories transferred from short-term to long-term storage? Systems-level memory consolidation is thought to be dependent on the coordinated interplay of cortical slow waves, thalamo-cortical sleep spindles and hippocampal ripple oscillations. However, it is currently unclear how the selective interaction of these cardinal sleep oscillations is organized to support information reactivation and transfer. Here, using human intracranial recordings, we demonstrate that the prefrontal cortex plays a key role in organizing the ripple-mediated information transfer during non-rapid eye movement (NREM) sleep. We reveal a temporally precise form of coupling between prefrontal slow-wave and spindle oscillations, which actively dictates the hippocampal-neocortical dialogue and information transfer. Our results suggest a model of the human sleeping brain in which rapid bidirectional interactions, triggered by the prefrontal cortex, mediate hippocampal activation to optimally time subsequent information transfer to the neocortex during NREM sleep.

## Abstract (web summary)

How are memories transferred from short-term to long-term storage? Here, the authors show that during deep (NREM) sleep, the prefrontal cortex initiates rapid, bidirectional interactions to trigger information transfer from the hippocampus to the neocortex.

## Introduction

Systems-level memory consolidation theory posits that initial memory encoding is supported by the hippocampus, but that overtime, memory representations become increasingly dependent upon the neocortex^1^. A prevailing view is that such information transfer occurs, in part, during NREM sleep^1^. Specifically, the hippocampus generates sharp-wave ripples (~80–120 Hz in humans), which initially facilitate the reactivation of recently learned information^2,3^. Ripples do no occur in isolation, but appear tightly coupled to both slow oscillations (SO; <1.25 Hz) and sleep spindles (12–16 Hz)^4–7^, which are thought to mediate synaptic plasticity^1,8,9^. In particular, cortical SOs govern the precise temporal coordination of sleep spindles and predict overnight hippocampus-dependent memory formation^4,10^.

Currently, the majority of evidence supporting this theory stems from invasive recordings in rodents, given the difficulty of imaging the human hippocampus with a sufficiently high temporal resolution needed to detect ripples. Furthermore, it is unclear how findings obtained in rodents relate to humans, given the different anatomical organization, in particular in the prefrontal cortex (PFC)^11,12^.

One influential model suggests that hippocampal ripple activity is associated with reactivation of newly acquired information^13^, which strengthens the mnemonic representation, before it is transferred to the neocortex^14^. Ripples are thought to trigger subsequent cortical SOs and spindles, which promote neuroplasticity to facilitate long-term storage^15,16^. Contrary to this model, several recent reports indicated that cortical activity might actually precede hippocampal ripples^17–19^, bringing into question who is the main driver of these interactions in support of memory consolidation.

While several lines of research converged on the notion that the coupling of SOs, spindles and ripples is important for memory consolidation^4,5,10,16^, it is unclear if coupled SO-spindles simply index preceding hippocampal processing, or instead, if they play a functional role in organizing hippocampal activity^1,9^. The latter finding would indicate cortical control of hippocampal reactivations, one that may advantageously insure that the neocortex receives information at an optimal time point when information transfer^20,21^ and plasticity dynamics^22,23^ are maximal.

By directly recording from the hippocampus and PFC, we demonstrate that prefrontal SO-spindle coupling initiates a bidirectional processing cascade. The quality of the SO-spindle coupling predicts hippocampal dynamics and shapes the hippocampal-neocortical information transfer in the human brain.

## Results

**Fig. 1:**
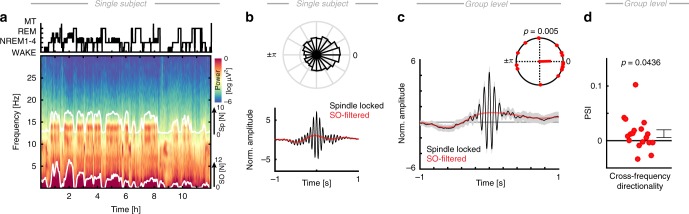
Sleep architecture and coupled NREM oscillations. **a** Top: hypnogram of a single subject (MT = movement time). Bottom: corresponding multi-taper spectrogram with superimposed number of detected SO and sleep spindle events (white solid lines; 5 min averages). **b** Top: normalized circular histogram of detected spindle events relative to the SO phase. Note the peak around 0° (circular mean = 15.27°, i.e. after the SO peak). Bottom: peak-locked sleep spindle average across all detected events in NREM sleep (black). Low-pass filtered events (red) highlight that the sleep spindles preferentially peaked during the SO ‘up-state’. **c** Peak-locked spindle grand average (black; *N* = 18; scalp EEG) superimposed with the SO low-pass filtered signal (red). Inset: spindles were significantly nested in the SO peak (V-test: *p* = 0.0050; red dots depict individual subjects). **d** Directional SO-spindle coupling as measured by the phase-slope index (PSI; mean ± SEM). SO phases significantly predicted spindle amplitudes (one-tailed *t*-test: *p* = 0.0436). Red dots depict individual subjects

We combined intracranial recordings from the human medial temporal lobe (MTL) and prefrontal cortex (PFC) in epilepsy patients with scalp EEG recordings to test if coupled SO-spindle activity mediates hippocampal-neocortical information transfer during a full night of sleep (Fig. 1a, see also Supplementary Table 1). In 18 subjects, we simultaneously obtained frontal scalp EEG and MTL intracranial EEG, while a subset of 15 patients also had intracranial coverage of three PFC subregions (dlPFC, mPFC, and OFC). The majority of MTL electrode contacts were placed in the hippocampus (CA1, CA3/DG, and Subiculum), but we also assessed adjacent contacts in entorhinal cortex (ERC), perirhinal cortex (PRC), and parahippocampal gyrus (PHG) given that ripples have been observed in both the ERC^24^ as well as the PHG^6^. We collectively refer to this group as ‘MTL’ electrodes throughout the manuscript, unless we found effects that were specific to the hippocampus proper. Furthermore, we utilized bipolar referencing to minimize effects of volume spread on our connectivity analyses; hence, most contacts contained hippocampal activity after re-referencing (~70%; Supplementary Table 2).

### Analysis strategy

Interictal epileptic discharges were excluded from all analyses (Supplementary Fig. 1)^7,25^. We focused on the most anterior available scalp electrode (typically Fz; subsequently referred to as ‘EEG’). This approach was in line with previous reports, which also utilized scalp electrodes as a surrogate of prefrontal activity^6,7^. We then performed similar analyses using only the intracranial PFC contacts (subsequently referred to as ‘PFC’) to investigate e.g. the spatial extent of network interactions and information transfer or directional cross-frequency coupling (CFC), which cannot be obtained from a single scalp electrode. Where applicable, we show both the EEG-MTL as well as PFC-MTL interactions to indicate that frontal EEG sensors indeed reflect a valid surrogate of the underlying population activity as measured by intracranial EEG.

**Fig. 2:**
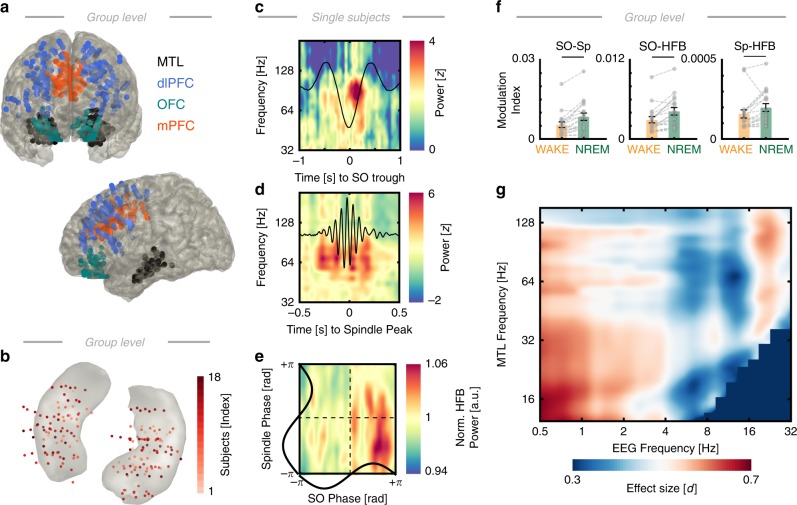
Intracranial electrodes and hierarchical nesting of sleep oscillations. **a** Schematic depiction in MNI space of the intracranial electrode placement in the medial temporal lobe (MTL; in black) and three PFC regions-of-interest (ROI) across the subset of subjects (*N* = 15) with simultaneous coverage of all three prefrontal ROIs (dlPFC: *n* = 167; OFC: *n* = 62; mPFC: *n* = 116). **b** Schematic depiction of MTL contacts (*n* = 184 electrodes in 18 subjects) on a hippocampal mesh in MNI space. Note that electrode size has been rescaled for display purposes and does not reflect the original electrode size. Precise electrode localizations were determined in native space prior to warping to MNI space. **c** Single subject time-frequency spectrogram from one MTL channel relative to a rescaled trough-locked cortical average SO (black). Note the circumscribed increase in high-frequency-band activity (~80–100 Hz) just prior to the SO peak. **d** Single subject time-frequency spectrogram from one MTL channel relative to a rescaled peak-locked cortical spindle average (black). High-frequency band activity exhibited several bursts during spindle events. **e** Single subject, single electrode example of simultaneous nesting of HFB power in the cortical SO peak (*x*-axis) and the cortical spindle trough (*y*-axis). **f** Cross-frequency coupling analysis indicated increased SO-Spindle, SO-HFB, and Spindle-HFB coupling during NREM sleep (gray dots depict individual subjects; mean ± SEM). **g** Full comodulogram (wake vs. NREM) depicting the effect size of the difference (Cohen’s d)

**Fig. 3:**
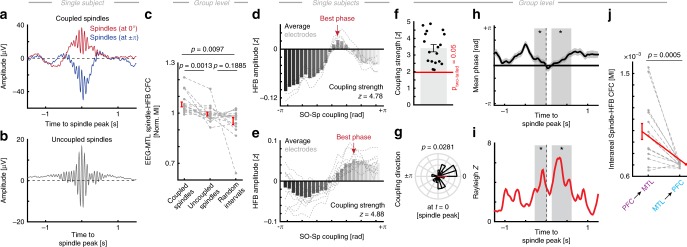
Precise cortical SO-spindle coupling phase predicts MTL HFB activity. **a** Single subject average of all coupled spindles that were nested in the SO peak (in red) and trough (in blue). **b** All uncoupled spindles, i.e. that did not coincide with a separate SO detection. **c** Cortical spindle to MTL HFB phase amplitude coupling for coupled and uncoupled spindles as well as event-free random NREM intervals (normalized by the individual mean to reduce inter-subject variability). **d** Single subject HFB activity in the MTL as a function of the precise cortical SO-Spindle phase at the time of the spindle peak. Bars depict the average across electrodes; gray lines depict single electrodes. The red arrow indicates the SO-spindle coupling phase where the highest HFB amplitude was observed (~0°). **e** Different single subject example. Same conventions as in panel **d**. Note that HFB peaked after the SO peak (>0°). **f** Coupling strength: in every subject, we assessed the coupling strength by comparing the observed circular-linear correlation between SO-spindle coupling phase and HFB amplitude to a surrogate distribution. The correlation coefficient was then *z*-scored relative to a phase-shuffled distribution of correlation coefficients and considered significant at *z* = 1.96 in individual subjects (corresponding to a two-tailed *p* < 0.05; red solid line). The gray bar depicts the mean across subjects; black dots depict individual subjects. We observed significant HFB modulations in 18/18 subjects (mean *z* = 3.40, *p*_group_ = 0.0007). **g** Preferred coupling phase: circular histogram of the best phase (panels **d** and **e**) where HFB peaked in individual subjects. At the group level, HFB amplitude was highest during the spindle when the spindle was nested in the SO peak (mvl = 0.44). **h** Preferred coupling phase across time (circular mean ± circular SEM). We repeated the analysis depicted in panel **f** for all time points (±1.25 s around the spindle peak). Shaded bars depict cluster-corrected significant Rayleigh tests (cluster *p* < 0.001) for circular non-uniformity (test for phase consistency at the group level), indicating that a preferred phase across subjects emerged around the spindle peak (cluster 1: −0.3 to 0.04 s; cluster 2: 0.12 to 0.60 s); hence, the effect was transient and not sustained. **i** Rayleigh Z and significant clusters as described in panel **h**. **j** Directional CFC analysis (gray dots depict individual subjects; mean ± SEM) indicating that the PFC spindle phase was predictive of hippocampal HFB, but not vice versa (rank sum test: *p* = 0.0005). All error bars indicate the mean ± SEM

**Fig. 4:**
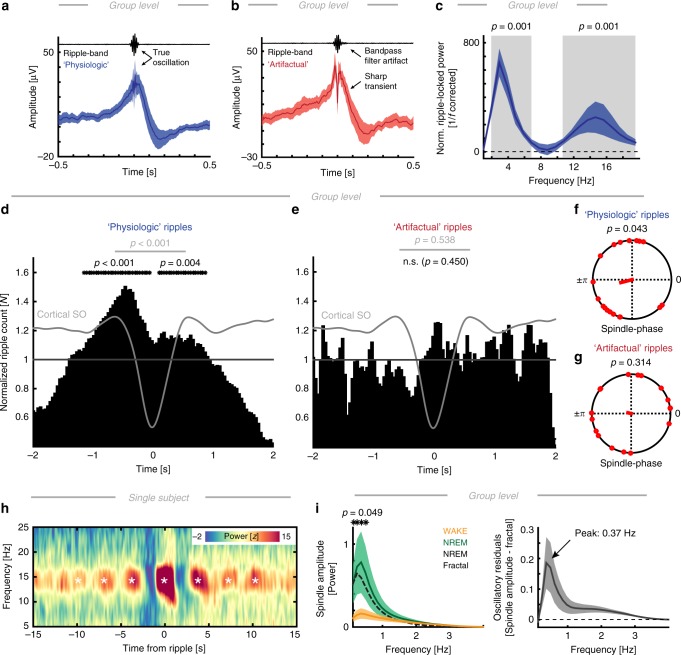
Physiological and artifactual ripple activity in the MTL. **a** Ripple-locked grand average (blue; *N* = 18) and the band-bass filtered signal (black; mean ± SEM). **b** Artifactual ripple events grand average (red) that are characterized by sharp transients that are artificially rendered sinusoidal by band-pass filtering (black). **c** 1/f-corrected power spectrum (mean ± SEM; irregular resampling) in the MTL during a ripple event exhibits two distinct oscillatory peaks: in the delta and spindle frequency range. Gray shaded areas indicate significant oscillatory activity (cluster test: *p* = 0.001). **d** Histogram of ripple occurrence relative to the cortical SO as detected at scalp level. Note ripple occurrence is enhanced during the cortical upstate (i.e., the SO peak, first cluster: *p* = 0.0010, *d* = 2.82; second cluster: *p* = 0.0040, *d* = 1.42), but significant more ripples occur prior to the downstate (in gray; 400 ms window around the SO peaks, paired *t*-test: *t*_17_ = 5.01, *p* < 0.0001, *d* = 1.88). **e** This relationship was not present for artifactual ripple events, which occurred at random (smallest cluster *p* = 0.450; no peak differences: *t*_17_ = −0.63, *p* = 0.5381, *d* = 0.27). **f** Physiologic ripples were preferentially nested in the trough of the cortical spindle oscillation (V-test against ± pi: *v* = 5.13, *p* = 0.0434, mvl = 0.29). **g** This tight temporal relationship was again not present for artifactual ripple events (*v* = 1.46, *p* = 0.3135, mvl = 0.08). **h** Single subject ripple-locked EEG spectrogram indicates the presence of cortical spindle oscillations during a hippocampal ripple. Note that activity in the spindle range can be observed before and after the ripple (every ~3 s; asterisks indicate elevate spindle power). **i** Left Spectral analysis of the spindle amplitude (green), its fractal (1/f estimate; black dashed line) as obtained from irregular resampling as well as the wake condition as a control (absence of spindle activity; cluster test: *p* = 0.0490, *d* = 0.49). Right: after subtraction of the fractal component from the NREM spectrum, we observed a distinct oscillatory peak indicating a rhythmic comodulation at ~0.37 ± 0.03 Hz (median ± SEM; sign test against zero: *p* < 0.0001; *d* = 0.70). All error bars indicate the mean ± SEM

**Fig. 5:**
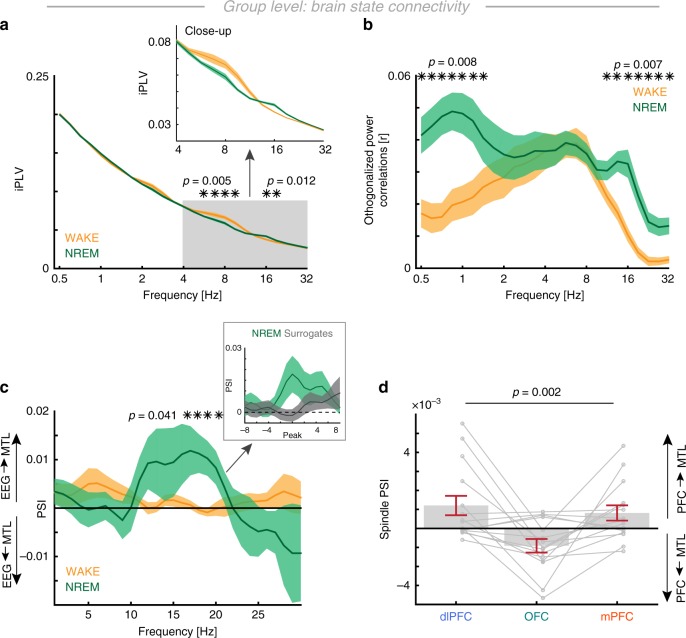
PFC-MTL connectivity and directionality. **a** Phase locking analyses revealed increased spindle phase coupling during NREM sleep (cluster tests: *p* = 0.0120, *d* = 1.37), but no elevated SO synchrony (*p* = 0.3087, *d* = 0.69). During wakefulness, we observed enhanced alpha-band coupling (*p* = 0.005, *d* = 0.83). **b** Power correlations (mean ± SEM) between scalp EEG and hippocampus revealed differences in amplitude couplings during NREM sleep (SO cluster: *p* = 0.0080, *d* = 1.07; spindle cluster: *p* = 0.0070, *d* = 1.20). **c** State-dependent directionality analysis indicates significant EEG to MTL influences (phase-slope index; mean ± SEM) in the spindle-band during NREM sleep (cluster test: *p* = 0.0410, *d* = 0.84). Inset: given the inter-subject variability in the precise spindle peak frequency, we also compared the peak-aligned (14.9 Hz ± 2.7 Hz; mean ± SD) PSI estimates to a surrogate distribution (gray), which confirmed the original observation (*t*-test: *t*_17_ = 2.16, *p* = 0.0452, *d* = 0.69). **d** Directional spindle coupling for every PFC ROI (lower panel; RM-ANOVA: *F*_1.57,21.92_ = 9.33, *p* = 0.002, *η*^2^ = 0.25) indicating a stronger modulatory influence from both the dlPFC and the mPFC but not the OFC. All error bars indicate the mean ± SEM

**Fig. 6:**
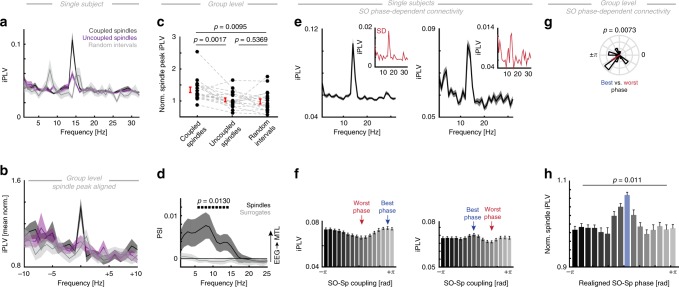
Precise SO-spindle coupling phase modulates MTL-PFC connectivity. **a** Single subject example of EEG-MTL connectivity calculated separately for coupled spindles (black), uncoupled/isolated spindles (purple), and random event-free intervals (gray). Event numbers were equated by a bootstrapping procedure. Note the distinct peak in the spindle band. **b** We re-aligned power spectra to the individual spindle peak (14.5 ± 2.6 Hz; mean ± SD) and mean-normalized the aligned spectra to minimize inter-subject variability. **c** We then extracted the normalized EEG-MTL iPLV peak values for all events. A repeated measures ANOVA revealed a significant main effect (*F*_1.49,25.27_ = 7.93, *p* = 0.0043, *η*^2^ = 0.21; *p*-values from post-hoc pair-wise comparisons are depicted). We found significantly more inter-areal connectivity for coupled than for uncoupled spindles or random event-free intervals. **d** Directionality analysis during spindle events revealed a significant influence from prefrontal EEG spindles to the MTL (dark gray) as compared to a surrogate distribution (light gray). **e** Two single subject examples of event-locked spindle connectivity as a function of the precise coupling phase (mean ± SEM). Upper: the spindle band exhibits a strong peak in the spectrum and also peaks in the standard deviation spectrum (inset in red), thus, indicating that the spindle band exhibits the largest variance. **f** Spindle-band connectivity binned as a function of the precise coupling phase revealed strong non-uniform distributions. We highlighted the worst (lowest synchrony; red) and best (highest synchrony; blue) phase. **g** Anti-phasic relationship between best and worst connectivity phase (EEG-MTL: V-test against ±π: *v* = 7.33, *p* = 0.0073, mvl = 0.49). **h** Realigned (to the best phase) grand average spindle (12–16 Hz) iPLV histogram reveals a non-uniform relationship (mean ± SEM) between SO-Spindle coupling and EEG-MTL phase locking. Note we excluded the central aligned phase bin from subsequent testing (RM-ANOVA: *F*_5.23,88.83_ = 3.13, *p* = 0.011, *η*^2^ = 0.15). All error bars indicate the mean ± SEM

**Fig. 7:**
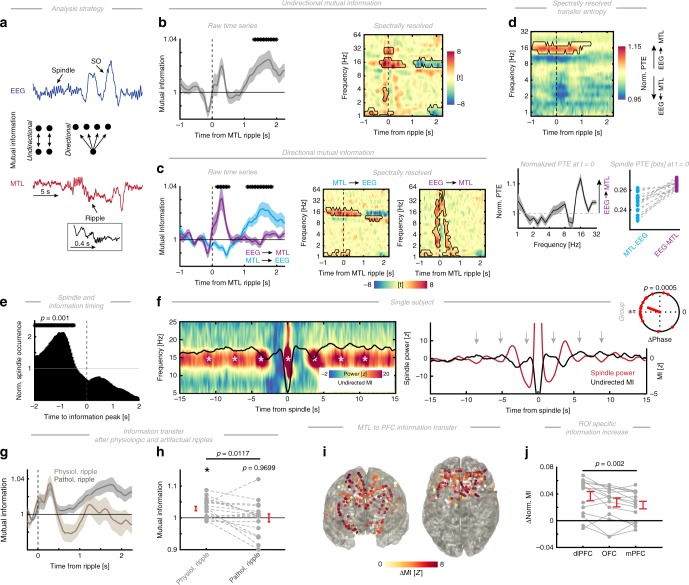
Ripple-mediated information transfer. **a** Illustration of the information analysis strategy. A single EEG trace featuring SOs and spindles (in blue) and the corresponding MTL trace (in red) are depicted relative to a ripple event (see close-up; in black). Undirectional information was calculated by a moving time-window approach (center left), while directional information was calculated by keeping one window centered on the MTL ripple time point (center right; here fixed MTL window to moving EEG window; EEG to MTL information was calculated accordingly). **b** Left: time-resolved Mutual Information (MI) relative to the hippocampal ripple event (mean ± SEM). We detected enhanced MI between the hippocampus and prefrontal cortex ~1 s after the ripple event (1.3–2.0 s; cluster test: *p* = 0.0040, *d* = 1.06), but also observed two non-significant clusters (positive cluster from 0.25 to 0.35 s, *p* = 0.0849; negative cluster from −0.15 to −0.10 s, *p* = 0.1349). Right: spectrally resolved undirected mutual information. Significant (two-tailed *p* < 0.05) deviations from the average MI. Prior to ripple onset, mutual information was enhanced in the spindle and SO/delta range, which after 1 s dropped. **c** Left: time-resolved MI relative to the ripple event. We disentangled the information flow from prefrontal EEG to the MTL (purple) and vice versa (cyan). A significant cluster was observed after the ripple (cluster test: negative cluster from 0.15 to 0.50 s; *p* = 0.0130, *d* = 1.31), indicating increased information flow from the prefrontal EEG to the MTL. We found a second significant cluster where MI increased after ~1 s, which mainly reflected MTL to prefrontal EEG information flow (positive cluster from 1.1 to 1.85 s; *p* = 0.001, *d* = 1.08). Center/right: frequency- and directionality-resolved information flow. Outlined areas (black) reflect significant clusters (*p* < 0.05), where information flow was enhanced relative to the mean. Center: frequency-specific information flow from the MTL to neocortex was primarily increased in the SO (<2 Hz) and spindle-bands (~16 Hz). Right: information flow from the prefrontal EEG to MTL was not frequency-specific. **d** Upper panel: phase transfer entropy between the prefrontal EEG and the MTL (>1) is stronger than in the opposite direction (<1), in particular in the spindle-band (from −1–1.4 s; cluster test: *p* < 0.0010, *d* = 2.02). Lower left: excerpt during the ripple (*t* = 0) of the normalized PTE spectrum highlights the peak at ~16 Hz. Lower right: single subject observations in the spindle-band during the ripple. **e** Normalized spindle occurrence relative to the information peak. Spindle occurrence was significantly enhanced prior to the information peak (−2 to −0.5 s; peak time −0.91 s; cluster test: *p* = 0.001, *d* = 3.37). **f** Left: spindle-locked time-frequency representation of a single subject over 30 s highlights the slow (~0.4 Hz) pattern in spindle power. Superimposed is the rescaled, time-resolved undirected MI between the MTL and scalp EEG (black). Right: average spindle power (red; demeaned) and undirected MI (black; *z*-scored) indicating an anti-phasic relationship (gray arrows indicate MI peaks that coincide with spindle troughs). Note that the y-axis is truncated at around 0 to highlight the side lobes. Inset: group-level results revealing a statistically significant anti-phasic relationship between spindle power and MI between the MTL and frontal EEG sensors. **g** MI was enhanced only after a physiologic ripple as compared to an artifactual ripple event. Given that artifactual ripples were noisy and hence, variance was increased, we averaged in the time domain (cluster in panel **a**) prior to statistical testing. **h** MI was enhanced only after a physiologic ripple as compared to an artifactual ripple event (paired *t*-test: *t*_17_ = 2.82, *p* = 0.0117, *d* = 0.70). Note that artifactual ripples were also not significantly different from the baseline (paired *t*-test: *t*_17_ = −0.04, *p* = 0.9699, *d* = 0.01), thus, indicating that no information was transferred. The asterisk indicates that physiologic ripples were different from baseline as tested in panel **a**. **i** Topographical depiction of significant MI increases. All electrodes that showed a significant MI increase (*z* > 1.96, uncorrected *p* < 0.05) are color-coded according to the *z*-score, while electrodes without significant modulation are depicted in white. Note, the increase in MI was widespread and not restricted to a circumscribed cortical region. **j** MI increases per prefrontal sub-region (RM-ANOVA: *F*_1.64, 23.01_ = 9.13, *p* = 0.0020, *η*^2^ = 0.05). The strongest increase was observed in the dlPFC. All error bars indicate the mean ± SEM

To effectively combine scalp and intracranial EEG, we adopted the following analysis strategy: First, we replicated and extended our recent observation that prefrontal SOs shape spindle activity (Fig. 1b–d)^10^. Note that all SOs and spindle events were detected at the prefrontal EEG electrode. Second, we established the relationship between the activity at scalp EEG level and all available MTL electrodes (Fig. 2). We utilized two complementary approaches: State-dependent (based on continuous 30 s segments as defined by the hypnogram) as well as event-related (based on automatic event detection) analyses. Third, we dissected the precise relationship between cortical spindle and MTL high-frequency band (HFB; 70–150 Hz) activity (Fig. 3), which captures both non-oscillatory broadband as well as oscillatory ripple events. Subsequently, we investigated whether the coupled HFB signatures reflected ripple oscillations (Fig. 4) and how those were precisely related to distinct cortical events, such as SOs and spindles. Having established that these three cardinal NREM oscillations dynamically interact during sleep, we then investigated the overall network connectivity as a function of brain state (Fig. 5) as well as in an event-related approach (Fig. 6) to further investigate the precise role of SO-spindle coupling on inter-areal communication. Finally, we quantified interregional information transfer relative to the ripple events (Fig. 7) that have been implicated in information reactivation, replay, transfer, and consolidation^2,3,15^.

### Cortical SO-spindle coupling shapes MTL HFB activity

We first detected SO and spindle oscillations in scalp EEG (Fig. 1) using established algorithms^7,10^. We utilized multi-taper spectral analyses^10,26^ to visualize spectral dynamics throughout the night (Fig. 1a). Event detection closely tracked spectral sleep signatures over a whole night of sleep. For every participant, we then determined the precise SO phase during the spindle peaks. We found significant (*p* < 0.05; Rayleigh test) non-uniform distributions in 14/18 subjects (Fig. 1b; *p* = 0.0154; one-tailed Binomial test; mean Rayleigh *Z* = 25.56 ± 6.32; resultant vector length: 0.17 ± 0.02, mean ± SEM). Spindles were preferentially nested in the SO peak (Fig. 1c; V-test against 0°: *v* = 7.73, *p* = 0.0050, mvl = 0.43; coupling phase: 1.94° ± 14.42°, circular mean ± SEM). We further quantified the directional influence using the phase-slope index^27,28^ (PSI) of SOs on spindles as reported previously (Fig. 1d)^10^. We again found that SO predicted spindle activity as indicated by a positive PSI (0.0128 ± 0.0071; mean ± SEM; one-tailed *t*-test against 0: *t*_17_ = 1.81, *p* = 0.0436, *d* = 0.61), which correlated negatively with age (Supplementary Fig. 2)^10^.

Next, we assessed how MTL activity (for electrode placement see Fig. 2a, b and Supplementary Table 2) was modulated as a function of SO and spindle activity. We found significant power modulations in the high-frequency band (HFB; 70–150 Hz), which captures both ripple oscillations as well as multi-unit spiking activity^29,30^, in the MTL relative to cortical SOs (Fig. 2c) and spindles (Fig. 2d). In particular, we found evidence in line with previous reports^6,7^ that maximal HFB activity was nested in the cortical SO peak and cortical spindle trough (Fig. 2e, see also below). Next, we calculated state-dependent cross-frequency coupling estimates using the modulation index^7,31^ between the three cardinal NREM frequency bands (Fig. 2f). We found significantly stronger SO-spindle (paired *t*-tests: *t*_17_ = −4.55, *p* = 0.0003), SO-HFB (*t*_17_ = −4.81, *p* = 0.0002) and Spindle-HFB coupling (*t*_17_ = −2.32, *p* = 0.0328) during NREM sleep. This finding was further corroborated by a full comodulogram, thus, replicating and confirming previous reports using different spectral estimates (Fig. 2g)^7^.

To determine how these three signatures dynamically interact within the human brain, we examined how hippocampal HFB activity is modulated by the precise cortical SO-spindle coupling phase. We first identified coupled (Fig. 3a) and uncoupled (Fig. 3b) spindles. Spindles were classified as coupled when a separate SO was detected in the same interval (±2.5 s; reflecting ± 2 SO cycles). We also extracted the precise SO coupling phase for every spindle (see Fig. 3a for spindles at 0° and ±180°). Next, we compared the inter-areal (EEG-MTL) spindle-HFB coupling between coupled, uncoupled and random, event-free NREM intervals (Fig. 3c). We found that inter-areal spindle-HFB coupling differed significantly (repeated measures ANOVA: *F*_1.30,22.15_ = 6.38, *p* = 0.013, *η*^2^ = 0.27). Post-hoc *t*-test indicated that coupled spindles exhibited more inter-areal CFC than uncoupled spindles (*t*_17_ = 3.82, *p* = 0.0013, *d* = 1.04) or random intervals (*t*_17_ = 2.91, *p* = 0.0097, *d* = 1.30). Uncoupled spindles and event-free random intervals did not differ significantly (*t*_17_ = 1.37, *p* = 0.1885, *d* = 0.59). Note, this result was independent of the chosen time window for co-occurrence detection (9 linearly spaced bins between 0.5 and 2.5 s; 2 × 2 RM-ANOVA with factors spindle coupling and time bin: Coupled spindles exhibited more inter-areal CFC: *F*_1,17_ = 15.42, *p* = 0.001, *η*^2^ = 0.39. No significant impact of factor time bin: *F*_2.40,40.75_ = 2.88, *p* = 0.059, *η*^2^ = 0.01; no significant interaction: *F*_2.90,49.31_ = 1.03, *p* = 0.384, *η*^2^ = 0.01). Furthermore, coupling estimates can be confounded by differences in oscillatory power as well as event numbers. Here we observed no differences in power between coupled and uncoupled spindles (*t*-test: *t*_17_ = 1.08, *p* = 0.2969) and stratification of trial numbers (50 repetitions) confirmed that inter-areal CFC of coupled spindles was significantly enhanced as compared to uncoupled spindles (*t*-test: *t*_17_ = 4.09, *p* = 0.0008).

Next, we sought to determine if not only the co-occurrence of SOs and spindles modulates HFB power, but if the precise temporal relationship fine-tuned HFB activity in the MTL. Therefore, we calculated the average HFB power in the MTL as a function of the precise SO-spindle coupling phase in 24 bins. Figure 3d, e highlights two single subject examples. Coupling strength was quantified in every subject as the circular-linear correlation coefficient between the coupling phase and the HFB amplitude, which was *z*-scored relative to a phase-shuffled surrogate distribution (1000 repetitions). We found significant coupling in 18/18 subjects (Fig. 3f; *z* > 1.96 corresponds to a two-tailed *p* < 0.05; mean *z* = 3.40, which corresponds to a group *p* = 0.0007; see also Supplementary Fig. 3). Comparable results were obtained when we utilized a RM-ANOVA with factor bins in all subject (significant at *p* < 0.05 in 15/18 subjects; Binomial test: *p* = 0.0075; *η*^2^ = 0.38 ± 0.06, mean ± SEM). Note that the precise phase bin where HFB amplitude peaked varied across subjects (red arrows in Fig. 3d, e). However, at the group level we found that HFB amplitude was significantly stronger coupled when the spindle peaked during the SO up-state (Fig. 3g; −1.00° ± 14.28°, circular mean ± SEM; Rayleigh test: *p* = 0.0281, Rayleigh *Z* = 3.49, mvl = 0.44).

Next, we investigated whether this triple coupling emerged in a temporally precise manner. Therefore, we repeated the analysis for all time points around the spindle peak (±1.25 s) and extracted the preferred phase for every subject at every time point (Fig. 3h). Using cluster-corrected Rayleigh tests, we found that a preferred coupling phase at the group level emerged around the spindle peak (1st cluster: −0.30 to 0.04 s, *p* < 0.001, mvl = 0.46; 2nd cluster: 0.12–0.60 s, *p* < 0.001, mvl = 0.51), indicating a temporal selective triple cortical-MTL coupling during the SO-spindle complex. Finally, we tested the directionality of the spindle-HFB coupling. Given that HFB cannot be reliably extracted from scalp EEG, we restricted this analysis to the 15 subjects with simultaneous intracranial PFC and MTL coverage. We calculated the inter-areal CFC between spindle phase and HFB amplitude for all PFC-to-MTL and MTL-to-PFC pairs (Fig. 3j) and observed significantly stronger directional coupling from the PFC-to-MTL than vice versa (Wilcoxon rank sum test, given the unequal variance: *z* = 3.48, *p* = 0.0005, *d* = 1.50).

Taken together, our analyses demonstrated that MTL HFB activity became selectively coupled to spindles, when spindles were precisely nested in the SO peak. We conclude that the quality of cortical SO-spindle coupling predicts and coordinates MTL HFB dynamics.

### MTL HFB activity reflects coupled ripple oscillations

We have focused on HFB activity, which reflects both aperiodic broadband activity as well as oscillatory ripple events. Both signatures exhibit similar spectral characteristics. Therefore, we identified distinct ripple events during SO-spindle coupling in the time domain (Fig. 4a and Supplementary Fig. 4a–c)^7,24^. We then excluded sharp artifactual and epileptic transients, which can be mistaken as ripples in the frequency domain (Fig. 4b). For every subject, we determined the channel with the highest number of physiologic ripples (Supplementary Fig. 4d). Subsequently, we centered our ripple-locked analyses on this channel to minimize the impact of artifacts and the bipolar referencing (phase reversals as seen Supplementary Fig. 4c) in line with previous reports^7^.

In order to investigate if there were any other prominent rhythms during a ripple event in the MTL, we transformed ripple epochs (±2.5 s) into the frequency domain. After discounting the 1/f contribution by irregular resampling^10,32^, we found two distinct oscillatory peaks in the delta (~3 Hz, reflecting the hippocampal sharp wave; cluster-based permutation test: *p* = 0.001, *d* = 1.69) and spindle range (~14.5 Hz, *p* = 0.001, *d* = 0.91; Fig. 4c).

Next, we assessed the relationship of ripples to cortical SOs and spindles. We calculated the ripple occurrence relative to cortical SOs. We found that hippocampal ripple occurrence is enhanced during the SO peak (Fig. 4d; cluster-based permutation test: 1st cluster: *p* = 0.0010, *d* = 2.82; 2nd cluster: *p* = 0.0040, *d* = 1.42), particularly evident prior to the down-state (i.e. SO trough; 400 ms window around the SO peaks, paired *t*-test: *t*_17_ = 5.01, *p* < 0.0001, *d* = 1.88). No such relationship was observed for artifactual ripple events (Fig. 4e; smallest cluster *p* = 0.450; no peak differences: *t*_17_ = −0.63, *p* = 0.5381, *d* = 0.27). This is in line with the pattern that was observed for the HFB (Fig. 2c–e). Likewise, we found that ripples were preferentially nested in the trough of the cortical spindle oscillation (Fig. 4f; V-test against ±pi: *v* = 5.13, *p* = 0.0434, mvl = 0.29; see also to Fig. 2d, e). This tight temporal relationship was again not present for artifactual ripple-like events (Fig. 4g; *v* = 1.46, *p* = 0.3135, mvl = 0.08).

To investigate cortical dynamics relative to the ripple on longer time-scales, we calculated ripple-locked EEG time-frequency representations (±15 s) and observed a striking pattern in the spindle band, where spindle rhythmically re-occurred every 3–6 s (Fig. 4h). In order to quantify this effect, we calculated the spectral content of the state-dependent spindle amplitude by means of an FFT and used wakening as a control condition (absence of spindle activity). In a cluster-based permutation test, we found significantly enhanced power in the <0.5 Hz range (cluster-based permutation test: *p* = 0.0490, *d* = 0.49). We also estimated the 1/f contribution by means of irregular resampling (Fig. 4i, right panel), which further corroborated our finding that spindle amplitudes exhibit a slow, rhythmic comodulation over time (peak frequency: 0.37 ± 0.03; median ± SEM). Taken together, we identified oscillatory ripples events in the MTL, which exhibit a tight temporal relationship to cortical SOs and spindles on multiple timescales.

### Brain state dependent connectivity and directionality

We established that the three cardinal spectral signatures of NREM sleep form an oscillatory hierarchy. This concept further implies that band-limited neural oscillations establish and support communication channels across distant cortical regions^33,34^. To address this, we determined the frequency bands in which the cortex and the MTL were coupled during the NREM sleep. We first focused on brain state dependent connectivity, which was calculated in 30 s segments as defined by the hypnogram. Undirected connectivity was calculated using metrics that suppressed effects of volume spread in the cortical tissue. We assessed two different coupling modes: Phase synchrony as measured by the imaginary phase locking value (iPLV)^35^ and amplitude-envelope correlations as measured by orthogonalized power correlations^36^.

We found that phase synchrony was only elevated in the spindle band (Fig. 5a; cluster-based permutation test: *p* = 0.0120, *d* = 1.37) but interestingly not in the SO range (*p* = 0.3087, *d* = 0.69). In addition, we found a negative cluster that indicated enhanced theta/alpha coupling during wakefulness as compared to NREM sleep (*p* = 0.005, *d* = 0.83). To further elucidate whether there is a long-range interaction in the SO range, which might not be phase-specific, we also computed power correlations between the EEG and MTL. This analysis revealed significant differences in both the SO (Fig. 5b; cluster-based permutation test: *p* = 0.0080, *d* = 1.07) as well as the spindle range (*p* = 0.0070, *d* = 1.20) as compared to wakefulness. However, from the observed pattern it is unclear whether this reflects a selective increase during NREM sleep or if the effect is driven by a relative increase of theta/alpha coupling during wakefulness. Notably, connectivity profiles as obtained from scalp EEG mimicked the pattern when intracranial PFC electrodes were used for connectivity analyses (Supplementary Fig. 5a, b). These state-dependent phase synchrony profiles exhibited a pronounced 1/f drop-off and the numerical differences were small.

An important contributing factor to this effect was that only a few spindle events occurred in a given 30 s epoch, hence, event-related synchrony effects might be underestimated when data is averaged in long epochs. Therefore, we investigated event-related connectivity profiles (see Fig. 6). An important observation that emerged from this analysis is the absence of pronounced SO phase synchrony between the EEG and MTL, indicating that SOs—in contrast to spindles—might play a negligible role for direct inter-areal information transfer. This was further corroborated by a directional connectivity analysis using the PSI (Fig. 5c). We found a spindle-band specific directional influence from the EEG to the MTL as compared to wakefulness (cluster test: *p* = 0.0410, *d* = 0.84) as well as when compared to a surrogate distribution (inset Fig. 5c). A PFC sub-region analysis (*N* = 15 subjects; RM-ANOVA: *F*_1.57,21.92_ = 9.33, *p* = 0.002, *η*^2^ = 0.25) indicated that the main drivers were the dlPFC (*t*_14_ = 2.36, *p* = 0.0166, *d* = 0.86; post-hoc one-tail *t*-tests against 0) and mPFC (*t*_14_ = 2.04, *p* = 0.0306, *d* = 0.74), but not the OFC (*t*_14_ = −2.56, *p* = 0.9887). No directional coupling effects were observed in the SO range (Fig. 5c). In summary, state-dependent directed and undirected connectivity profiles indicate that spindles play a pivotal role mediating directional influences from prefrontal to MTL regions.

### Cortical SO-spindle coupling predicts PFC-MTL connectivity

These observations raise the question which role SOs play for inter-areal communication. We tested the hypothesis that the precise cortical SO-spindle coupling phase predicts the magnitude of the inter-areal phase synchrony in support of information transfer. First, we calculated phase synchrony estimates for coupled and uncoupled spindles as well as random event-free epochs (Fig. 6a, b; analogous to the analyses described in Fig. 3c). On the group level, we observed significant differences in synchrony (Fig. 6b, c; RM-ANOVA: *F*_1.49,25.27_ = 7.94, *p* = 0.0043, *η*^2^ = 0.21). Post-hoc *t*-tests revealed that coupled spindles were more synchronous than uncoupled spindles (*t*_17_ = 3.73, *p* = 0.0017, *d* = 1.00) and event-free intervals (*t*_17_ = 2.92, *p* = 0.0095, *d* = 1.05). In contrast, uncoupled spindles and random intervals did not differ (*t*_17_ = 0.63, *p* = 0.5369, *d* = 0.18). We also tested the directionality of the spindle coupling using the PSI (Fig. 6d) as compared to a surrogate distribution given the absence of spindle activity during wakefulness. We found significantly enhanced PSI values, indicating a directed influence from the prefrontal EEG to MTL electrodes (cluster test: *p* = 0.0130, *d* = 0.77), thus, replicating the effect as observed for state-dependent analyses (Fig. 5c).

Having established that coupled spindles exhibit more inter-areal synchrony, we then assessed the impact of the precise coupling phase (analogous to the analyses in Fig. 3d–g). We observed clear peaks in the connectivity spectra that were centered on spindles (Fig. 6e; ±2.5 s). Notably, peaks were also evident in spectra depicting the standard deviation across 16 phase bins (insets Fig. 6e). We assessed EEG-MTL spindle synchrony as a function of the SO-spindle coupling phase in every subject (Fig. 6f). Non-uniformity across the 16 bins was assessed using RM-ANOVAs. We found significant (*p* < 0.05, mean *η*^2^ = 0.32 ± 0.023; mean ± SEM) distributions in 18/18 subjects (two-tailed Binomial test: *p* < 0.0001). The best coupling phase was variable across subjects, but peaked on average after the SO up-state (108.85° ± 16.34°; mean ± SEM; mvl = 0.26), while the worst bin was closer to the down-state (−174.58° ± 16.89°; mvl = 0.22). Within the individual, best and worst phase were preferentially separated by ~180° (Fig. 6g; V-test: *v* = 7.33, *p* = 0.0073, mvl = 0.49). Given that we observed this variability on the best connectivity phase, we re-aligned the binned spindle synchrony to the bin centered at ~12° and mean-normalized individual distributions to reduce inter-subject variability. This bin was excluded from subsequent testing. We assessed the non-uniformity across the remaining 15 bins using a RM-ANOVA. We found significant synchrony differences as a function of the precise SO-spindle coupling phase (Fig. 6h; *F*_5.23,88.83_ = 3.13, *p* = 0.0109, *η*^2^ = 0.15).

Collectively, these findings establish that the precise SO-spindle coupling phase predicts the connectivity between prefrontal and MTL sites and is in line with the notion that spindles selectively establish a communication pathway between the MTL and neocortex.

### Ripple-mediated information transfer

In a final step, we investigated the interplay between spindles and ripple-mediated information transfer. Ripples during hippocampal replay have been proposed to reflect information package transfer^2,3^. To quantify information transfer, we calculated time-resolved mutual information (MI)^37^ between the EEG and MTL relative to MTL ripple events (for a schematic of the analysis strategy see Fig. 7a). In particular, we utilized undirectional (time window by time window analyses) as well as directional (ripple-centered fixed time window to all other time windows) MI analyses. We first calculated the undirectional MI between the EEG and MTL (Fig. 7b left panel and Supplementary Fig. 6a). We found that MI reliably increased ~1 s after a ripple (cluster test: *p* = 0.0040, *d* = 1.06), which is in line with the idea that information is being reactivated during a ripple for further processing. Albeit non-significant (non-significant positive cluster from 0.25 to 0.35 s, *p* = 0.0849; non-significant negative cluster from −0.15 to −0.10 s, *p* = 0.1349), we also observed an additional decrement just prior to the ripple, which could potentially reflect a cortical trigger event initiating the ripple (e.g. a spindle; Supplementary Fig. 6b).

To investigate which frequency bands carried information, we next spectrally decomposed the signal. We z-normalized every frequency band separately to account for the 1/f signal drop-off and performed no baseline correction to assess information prior to the ripple. Spectrally resolved undirected MI estimates (Fig. 7b, right panel) revealed significant information away from the mean in the spindle-band (cluster test: *p* < 0.0010, *d* = 1.63) and SO range (*p* = 0.0060, *d* = 1.17) already evident prior to the ripple event. Note, these clusters also involved the delta (~3 Hz; possibly reflecting delta waves or sharp-wave activity^7^) and beta-activity (~32 Hz; possibly reflecting a spindle harmonic^7^). Spectral techniques are known to give rise to temporal smearing due to the inherent time-frequency trade-off^34^. However, spectral decomposition offers the advantage that the signal is not mainly dominated by strong low-frequency components, which are particularly pronounced when analyzing data in the time domain due to the 1/f signal drop-off.

In order to test if this increase reflected ripple-specific information, we calculated time-resolved MI between a fixed time-window, which was centered on the MTL ripple (±0.2 s) and all other time points (Fig. 7c, left panel). To resolve directionality, we performed this fixed window analysis twice, either centered on the MTL or the EEG. This analysis revealed that the increase that occurred ~1 s after the ripple mainly reflected information transfer from the MTL to the prefrontal EEG (Fig. 7c; cluster test: *p* = 0.001, *d* = 1.08).

Critically, we also found a prominent earlier cluster indicating increased shared information between the EEG (at the time of the ripple event) and the MTL (following a ripple; cluster test: *p* = 0.0130, *d* = 1.31), supporting rapid bidirectional interactions between the prefrontal EEG and MTL after a ripple. To further characterize this effect, we calculated time-, frequency- and directionality-resolved MI (Fig. 7c, center and right panel). We observed that shared information between the MTL ripple and the prefrontal EEG was already enhanced prior to the ripple (Fig. 7c, center panel), in particular in the low-frequency (−0.9 to −0.25 s; <2 Hz; cluster test: *p* = 0.0110, *d* = 0.87) and spindle-band (−0.85 to 0.85 s; ~16 Hz; *p* < 0.0010, *d* = 0.73). Another cluster emerged later (1.1 to 1.9 s, ~11 Hz; *p* = 0.0050, *d* = 0.59). This observation further supports the idea that cortical SOs and spindles are predictive of MTL ripple activity (c.f. Figs. 3 and 4). The control condition (Fig. 7c right panel; information between a fixed EEG window and all other MTL time points) did not reveal a frequency-specific effect, but was broadly distributed and covered all frequency bins (three clusters; all *p* < 0.0240; mean *d* = 1.62). Note, that the moving window analysis is comparable to a non-linear lagged correlation analysis, but does not actually quantify frequency-specific information flow. Hence, we calculated transfer entropy as directional information-theoretical metric of information flow (Fig. 7d).

We found that phase transfer entropy was stronger from the prefrontal EEG to the MTL than vice versa in the spindle-band (~16 Hz; from −1 to 1.4 s around the ripple; cluster test: *p* < 0.0010, *d* = 2.02). We also observed prominent lower frequency components (<3 Hz; Supplementary Fig. 6c), which resemble the low-frequency effects observed in Fig. 7b, c, but these did not exhibit a preferred directionality, mimicking the observed pattern in Fig. 5a, c.

Having established these interactions, we also determined the delay between cortical spindle activity and information transfer (Fig. 7e). In line with the spindle-ripple interactions (Fig. 4) and ripple-information dependency (Fig. 7b, c), this analysis suggested that spindles preferentially occurred 0.5–2 s prior to the information peak as extracted from single-event traces (cluster test: *p* = 0.001, *d* = 3.37). Hence, ripple-mediated information could be detected at prefrontal sites after spindle offset, i.e., in between two spindles when the neocortex is relatively desynchronized, a neurophysiological state that maximizes information-processing capacities^1,9,21,37^. To further highlight this relationship, we calculated spindle-locked time-frequency representations (Fig. 7f; analogous to Fig. 4h), which reveal the anti-phasic between spindle power and MI. To quantify this effect, we calculated the average phase difference at ~0.4 Hz between spindle power and MI. At the group level (inset in Fig. 7f), we found strong non-uniform distributions with a preferred phase difference of (159.6° ± 11.7°, circular mean ± SEM; Rayleigh test: *Z* = 7.02, *p* = 0.0005, mvl = 0.62). These findings were further corroborated by the observation that no reliable information transfer occurred after an artifactual ripple (Fig. 7g, h; see also Supplementary Fig. 6d).

Finally, we assessed the spatial extent of this information transfer in the subset of subjects with intracranial PFC electrodes (*N* = 15; Fig. 7i). We found widespread ripple-mediated information increases, which were most pronounced in the dlPFC (Fig. 7j), but also significant in all other ROIs (paired *t*-test against 0; dlPFC: *t*_14_ = 5.35, *p* = 0.0001, *d* = 1.95; OFC: *t*_14_ = 4.12, *p* = 0.0010, *d* = 1.51; mPFC: *t*_14_ = 4.15, *p* = 0.0010, *d* = 1.52). Taken together, these findings are consistent with the idea that ripples mediate information transfer from the MTL to the neocortex^2,15,38^. The data described in Fig. 7I, j indicates widespread increments in shared information following a MTL ripple, not constrained to a single PFC sub-region. Importantly, we observed a clear temporal relationship between information peaks and spindle events (Fig. 7e, f), which might reflect distinct episodes of information reactivation (triggered by spindle synchrony) and information transfer in line with previous behavioral evidence^20^.

## Discussion

Our results demonstrate that precisely coupled cortical SOs and spindles modulate hippocampal dynamics in support of hippocampal-neocortical information transfer in the human brain. In particular, the quality of this coupling predicts inter-areal connectivity, triggers hippocampal ripple activity and shapes subsequent information transfer. Our findings describe two distinct cortical states during NREM sleep, which alternate every 3–6 s. First, we observed states of high synchrony where coupled SO-spindle complexes engage the MTL and trigger ripples (Figs. 3–6). Second, we also found states of low synchrony, where information flow from the MTL to the neocortex was maximized, but no prominent oscillatory activity was present (Fig. 7). This two-step organization might reflect an endogenous timing mechanism, which ensures that information arrives in the neocortex when processing capacities are optimized^20,22^. Taken together, our results suggest that synchronized sleep oscillations provide temporal reference frames for feed-forward and feedback communication and structure hippocampal-neocortical dynamics in space and time^4^.

Hippocampal-prefrontal pathways and their role for memory formation have been the studied extensively over the last few decades^4,7,15,16,39,40^. However, the majority of evidence stems from recordings in rodents, which exhibit the same prominent network oscillations, such as SOs, spindles and ripples, despite a dramatically different anatomical structure, in particular in the PFC^11,12^. A major contributing factor is that imaging the human MTL with high spatiotemporal resolution is challenging when using non-invasive methods^41^. Here we took advantage of recordings from patients who underwent invasive monitoring for seizure localization and were implanted with electrodes in different key nodes of the MTL-PFC network. While previous human intracranial studies demonstrated the presence of hippocampal ripples^24^ and the nesting of sleep oscillations^6,7^, these studies did not address the directionality of these interactions nor did they quantify information transfer or assess the contributions of distinct PFC sub-regions.

While memory research has focused on the hippocampus-dependent encoding of novel information, how this information is transferred to long-term storage is unknown. The discovery of neuronal replay provided an elegant mechanistic explanation of how mnemonic representations are strengthened, but this concept does not directly explain how information is subsequently transferred in large-scale networks^14^. Furthermore, it remains unclear how the different cortical nodes ensure that the receiving area is in a favorable state to process the reactivated information^40^.

Systems memory consolidation suggested a two-step process: Mnemonic reactivation is associated with hippocampal ripple activity that is tightly coupled to subsequent cortical SOs and spindles, which then mediate neuroplasticity to facilitate long-term neocortical storage^1–3,16^. This model received substantial empirical support over the last two decades^2,3,13,15,42,43^, but has been challenged recently^44–46^. In line with several of our observations (e.g. early post-ripple PFC-to-MTL information flow; Fig. 7c), several recent studies reported early cortical contributions preceding hippocampal involvement, thus, reflecting a promising avenue for future studies investigating interactions between the hippocampus and neocortex in support of memory formation^18,47–49^.

In particular, it had been observed that SOs, spindles, and ripples are precisely coupled to one another and form an oscillatory hierarchy, where spindles are nested in SOs and ripples are nested in spindles^5,7^. Crucially, several groups reported that this triple coupling predicts behavior^4,16^. For example, ripple-triggered electrical stimulation increased subsequent prefrontal SO-spindle coupling and recall performance^16^. Hence, some theories assumed that the hippocampus is the driver of this interaction and subsequent SOs and spindles mainly index hippocampal processing that facilitated neocortical processing^15,16^. We observed several data points that contradict the classic systems memory consolidation theory: first, not all signatures of information transfer are frequency-specific (e.g. Fig. 7c). Second, we found evidence for early cortical engagement around the ripple events, which is not in-line with classic systems consolidation theory. Given the recent observation of early cortical memory representations in humans^48,49^, we speculate that this could reflect a uniquely human network feature, requiring further explication using combined electrophysiology and behavioral testing tracking mnemonic representations^13^.

Notably, several other recent reports also implied an inverse directionality compared to classic theory^1,38,40^: SOs predict spindles, which in turn predict ripples. Several reports suggested that cortical activity might actually precede hippocampal reactivation^17–19^, none of which were carried out in humans. The present findings provide empirical evidence for this account in the human brain. Here we demonstrate that SOs shape spindles, which in turn trigger nested ripples. Crucially, the quality of cortical SO-spindle coupling directly predicts ripple-band activity as well as inter-areal synchrony. These findings provide clear evidence that the hippocampal-neocortical dialogue relies on rapid bidirectional communication, where the neocortex can mediate hippocampal information reactivation and subsequent transfer through neocortical-hippocampal-neocortical loops.

It had long been recognized that spindle activity predicts overnight memory retention, but the underlying mechanisms remained unclear^8^. In particular, spindle magnitudes or densities have commonly been used to assess their role for memory consolidation^50^. More recently, several reports indicated that not spindle activity per se, but spindle timing relative to the SO, determines the success of information reactivation and consolidation^7,10,51–54^. These findings were nicely paralleled by a two-photon calcium imaging study in rodents, which revealed that excitatory neural activity is amplified when spindles coincide with the SO peak, but not when they miss a narrow ‘window-of-opportunity’^22^. This pattern mirrored our observation that connectivity and inter-areal coupling is increased for coupled as compared to uncoupled spindles. This observation reveals that coupled spindles do not reflect one entity, but connectivity and coupling depend on the precise SO coupling phase^10,23^.

Furthermore, it had recently been observed that spindles exhibit a second-order temporal structure and rhythmically reoccur every 3–6 s^20^. We observed a highly comparable pattern for human hippocampal ripples. This has previously observed in rodents, albeit on a slower time-scale (~12 s)^5^. Several lines of research converged on the notion that spindles are associated with information reactivation, but that subsequent processing actually occurs ~1–2 s after a spindle^20,55^. In this state, the cortex is maximally desynchronized during NREM sleep and entropy and processing capacities are maximized^21^. These findings are consistent with recent evidence suggesting that cued memory reactivation during the spindle refractory period, i.e., at the peak information transfer, is detrimental for memory consolidation^20^. Our results provide empirical evidence and offer a mechanistic explanation for this behavioral observation. The present findings reveal that cortical spindles shape hippocampal ripples and subsequent information transfer from the MTL-to-PFC, which peaks 1–2 s after the spindle, i.e., during spindle refractoriness^9,20,55^. We speculate that additional sensory input during the inter-spindle interval might interfere with MTL-neocortical information transfer and thereby, explain the detrimental effects on memory by cue presentation during the spindle refractoriness^20^.

Seminal work by Steriade et al.^56,57^ indicated that spindles are mainly generated in the thalamus and in a cortico-thalamic loop. In the present study, we only recorded from locations that were actively explored for clinical purposes of seizure onset localization and did not involve the thalamus^41^. However, a recent rare human intracranial study that simultaneously recorded from the thalamus and the PFC, but not the MTL, reported that thalamic spindles are triggered by a neocortical down-state and are then back-projected to the neocortex where the spindle coincides with the next SO up-state^58^. It is plausible that neocortical SOs trigger thalamic spindles, and jointly provide a messenger mechanism to trigger hippocampal reactivation and transfer. Taken together, several lines of inquiry now indicate that frequency-specific bidirectional communication in large-scale networks during NREM sleep coordinates inter-areal information flow in support of long-term memory retention.

In summary, we established that bidirectional hippocampal-neocortical interactions support hippocampal information reactivation and transfer during NREM sleep. This dynamic process ensures that the neocortex receives mnemonic data at a temporally optimal physiological moment for information transfer and neuroplasticity^1,2,8,10^. Given clinical time constraints in this study, we did not obtain concomitant behavior. However, the current observed physiologic patterns are in accord with recent findings on how coupled SO-spindles support hippocampus-dependent memory consolidation^10^. These findings are of immediate clinical relevance, given that temporal dispersion of cortical sleep networks has been suggested to constitute a novel pathway of age- as well as disease-related cognitive decline^10,52,59–61^.

## Methods

### Participants

We obtained intracranial recordings from 18 pharmacoresistant epilepsy patients (35.61 ± 12.31 years; mean ± SD; 10 female) who underwent pre-surgical monitoring with implanted depth electrodes (Ad-Tech), which were placed stereo-tactically to localize the seizure onset zone. All patients were recruited from the University of California Irvine Medical Center, USA. Electrode placement was exclusively dictated by clinical considerations and all patients provided written informed consent to participate in the study. Patients selection was solely based on MRI confirmed electrode placement in the hippocampus. The study was not pre-registered. All procedures were approved by the Institutional Review Board at the University of California, Irvine (protocol number: 2014–1522) as well as by the Committee for Protection of Human Subjects at the University of California, Berkeley (Protocol number: 2010-02-783) and conducted in accordance with the 6th Declaration of Helsinki.

### Experimental design, data acquisition, and procedure

We recorded a full night of sleep for every participant. Recordings typically started around 8.00–10.00 pm and lasted for ~10–12 h (Supplementary Table 1). Only nights that were seizure-free were included in the analysis. Polysomnography was collected continuously.

### Sleep monitoring and data acquisition

We recorded from all available intracranial electrodes. In order to facilitate sleep staging based on established criteria, we also recorded scalp EEG, which typically included recordings from electrodes Fz, Cz, C3, C4, and Oz according to the international 10–20 system. Electrooculogram (EOG) was recorded from four electrodes, which were placed around the right and left outer canthi. All electrophysiological data was acquired using a 256-channel Nihon Kohden recording system (model JE120A), analog filtered at 0.01 Hz and digitally sampled at 5000 Hz.

### CT and MRI data acquisition

We obtained anonymized postoperative CT scans and pre-surgical MRI scans, which were routinely acquired during clinical care. MRI scans were typically 1 mm isotropic.

### Data analyses

Data analysis was carried out in MatLab 2015a (MathWorks Inc.), using custom code as well as functions from the EEGLAB toolbox^62^, the FieldTrip toolbox^63^, the CircStat toolbox^64^, Freesurfer and SPM12 as well as related toolboxes as described below^65^. In general, most analyses were carried out using custom code. In case we utilized specific equations, we provide these below. In addition, we used the following standard functions from toolboxes: Filtering (EEGLAB: eegfilt.m; or FieldTrip: ft_preprocessing.m). Preprocessing, spectral analyses and connectivity analyses were mainly carried out using FieldTrip (ft_preprocessing.m, ft_freqanalysis.m and ft_connectivityanalysis.m). Circular statistics were carried out in CircStat (Rayleigh test: circ_rtest.m, V-test: circ_vtest, mean vector length: circ_r, circular mean: circ_mean.m, circular SD: circ_std.m). Anatomical reconstructions using Freesurfer and SPM12 are in detail described here in tutorial format^66^. Code for using the phase-slope index^27^ (PSI) and irregular resampling^32^ (IRASA) is provided in the original publications.

### Electrode localization

Two independent neurologists visually determined all electrode positions based on individual scans in native space. For further visualization, we reconstructed the electrode positions as outlined recently^66^. In brief, the pre-implant MRI and the post-implant CT were transformed into Talairach space. Then we segmented the MRI using Freesurfer 5.3.0^67^ and co-registered the T1 to the CT. 3D electrode coordinates were determined using the Fieldtrip toolbox^63,66^ on the CT scan. Then we warped the aligned electrodes onto a template brain in MNI space to facilitate visualization on the group level. Note that the MNI reconstruction was only done for visualization purposes, but electrode localization was determined in native space. We assigned MTL electrodes to putative MTL subregions (CA1, CA3/DG, Subiculum, entorhinal cortex, perirhinal cortex, and parahippocampal gyrus) after visual inspection^68,69^. Therefore, bipolar pairs as reported mostly captured activity from more than one subfield.

### Polysomnography

All available artifact-free scalp electrodes were low-pass filtered at 50 Hz, demeaned and de-trended, downsampled to 400 Hz and referenced against the average of all clean scalp electrodes. EOGs were typically bipolar referenced to obtain one signal per eye. A surrogate electromyogram (EMG) signal was derived from electrodes in immediate proximity to neck or skeletal muscles, by high-pass filtering either the ECG or EEG channels above 40 Hz. Sleep staging was carried out according to Rechtschaffen and Kales guidelines by trained personnel in 30 s segments^70^ as reported previously^10,60^.

### EEG data

*Preprocessing:* Scalp EEG was demeaned, de-trended and locally referenced against the mean of all available artifact-free scalp electrodes. We applied a 50-Hz low-pass filter and down-sampled the data to 500 Hz. Scalp EEG analyses were typically centered on electrode Fz unless stated otherwise. In a subset of subjects (*N* = 5) Fz was not available and Cz was utilized instead of Fz.

In every subject (*N* = 18), we selected all available electrodes within and close to the hippocampus and entorhinal cortex, which were then demeaned, de-trended, notch-filtered at 60 Hz and its harmonics, bipolar referenced to its immediate lateral neighboring electrode and finally down-sampled to 500 Hz (ft_preprocessing.m). We retained all MTL channels that exhibited interictal epileptic spiking activity, but discarded noisy channels. In total, 15 out of 18 subjects had also simultaneous coverage of all three prefrontal sub-regions (dorsolateral (dlPFC), orbitofrontal (OFC), and medial (mPFC) prefrontal cortex). Again, all available contacts in these regions were included and the same preprocessing steps were applied. Then all resulting traces were manually inspected and noisy, epileptic and artifact-contaminated PFC channels were excluded.

For all inter-areal coupling and connectivity analyses, we calculated all pair-wise channel combinations before averaging across channels in a given ROI.

*Interictal epileptic discharge (IED) detection*: Prior to all analyses, we first detected IEDs using automated algorithms on all channels located in the MTL. We utilized two different algorithms, which led to comparable results, both in terms of the number of detected events as well as resulting waveform shapes (Supplementary Fig. 1). Hence, we utilized the first algorithm for all subsequent analyses. All cut-offs were chosen in accordance with recently published findings^7,25^ and were confirmed by two neurologists who visually verified the detected events.

*IED detection algorithm 1*^25^: The continuous signal was filtered front and backwards between 25 and 80 Hz (eegfilt.m) and the analytical amplitude was extracted from the Hilbert transform (hilbert.m) and then *z*-scored. Events were detected when this signal was 3 SD above the mean for >20 ms and <100 ms. The IED events were then time-locked to the peak and the epoch (±2.5 s) was considered as an artifact.

*IED detection algorithm 2*^7^*:* Here we considered three signals: the raw trace, an amplitude difference trace (amplitude difference between two time points) as well as a 200 Hz high-pass filtered version of the raw signal. We turned every trace into a *z*-score based on the individual stage-specific mean and SD. A time point was marked as artifactual when it exceeded at *z*-score of six in any of these traces or if the raw trace plus either the difference or the filtered trace simultaneously exceeded a threshold of 4. Consecutive segments were grouped together and time-locked to the IED peak.

*State-dependent spectral analysis:* To obtain a continuous time-frequency representation of a whole night of sleep (Fig. 1a) we utilized multitaper spectral analyses^26,71^ (ft_freqanalysis.m), based on discrete prolate slepian sequences. The raw data was epoched into 30-s-long segments, with 85% overlap (ft_redefinetrial.m). Spectral estimates were obtained between 0.5 and 30 Hz in 0.5 Hz steps. We utilized 29 tapers, providing a frequency smoothing of ±0.5 Hz. Note, different settings were used for all subsequent analyses to optimize the time-frequency trade-off.

*Event-locked intracranial spectral analyses* (Fig. 2c, d): We utilized multitaper spectral analyses based on discrete prolate spheroidal sequences in 21 logarithmically spaced bins between 32 and 181 Hz^71^. We adjusted the temporal and spectral smoothing to approximately match a 250 ms time window and ¼ octave frequency smoothing and baseline-corrected the values relative to the pre-event (−2 to −1.5 s) baseline.

*Ripple-locked intracranial spectral analysis:* After identification of ripple events in the time domain (see below), we transformed these events into the time-frequency domain using the multitaper method based on discrete prolate spheroidal sequences in 89 logarithmically spaced bins between 4 and 181 Hz^71^. We adjusted the temporal and spectral smoothing to approximately match a 250-ms time window and ¼ octave frequency smoothing and baseline-corrected the values relative to the pre-event (−2 to −1.5 s) baseline (Supplementary Fig. 4b). We utilized less frequency smoothing and a more fine-grained spectral resolution to facilitate detection of narrow-band high-frequency oscillations.

*Ripple-locked scalp EEG spectral analysis:* To observe ripple-related changes on a longer timescale (Fig. 4h), we extracted epochs (±30 s) around individual ripple events. For an optimal time-frequency trade-off, we utilized a single Hanning taper (window length 500 ms) and spectral estimates between 1–30 Hz were obtained in steps of 50 ms. We baseline-corrected these spectral estimates per frequency band by a *z*-score relative to a bootstrapped baseline distribution (−2 to −1s before ripple peak)^10^. The same approach was used for spindle-locked analyses (Fig. 7f). We removed both IEDs (Supplementary Fig. 1) and artifactual ripples (Fig. 4b) from all ripple-locked analyses, unless we specifically used artifactual ripples in the comparison (e.g. Fig. 7g, h).

*High-frequency band (HFB) activity:* We extracted the HFB activity by band-pass filtering the raw continuous time courses in eight non-overlapping 10 Hz wide bins ranging from 70 to 150 Hz (eegfilt.m) and applying a Hilbert transform to extract the instantaneous amplitude. Then every trace was separately normalized by a *z*-score after discounting the filter edge artifacts and all eight traces were averaged into one resulting HFB trace per channel (Fig. 3d, e). HFB power was then grouped according to the underlying SO and spindle phase (Fig. 2e; normalized on a mean of one).

*Amplitude modulations of ripple power* (Fig. 4c): We used irregular resampling^32^ (IRASA) to identify oscillatory components and separate them from broadband 1/f contributions. Ripple epochs were centered on the ripples (±2.5 s). IRASA resamples the neuronal signals by pairwise non-integer values (1.1–1.9 in steps of 0.05, as well as corresponding factors 0.9 to 0.1). This procedure slightly shifts the peak frequency of oscillatory signals by compressing or stretching the underlying signal. Note that the 1/f component remains stable. This procedure is then repeated in overlapping windows (window size: 1 s, sliding steps: 0.25 s; frequencies up to 20 Hz were estimated). Note resampling was always done in a pairwise fashion. We calculated the auto-power spectrum by means of a FFT after applying a Hanning window for all segments. Then all auto-spectra were median-averaged to obtain the 1/f component. Finally, the resampled 1/f PSD is subtracted from the original PSD to obtain the oscillatory residuals.

*Amplitude modulations of spindle power:* In order to test whether the amplitude of the spindle exhibits rhythmic modulations (Fig. 4h, i), we first band-pass filtered the continuous scalp EEG signal between 12 and 16 Hz and extracted the analytic amplitude from the Hilbert transform. Then we epoched this data into 30-s-long non-overlapping segments and disentangled oscillatory and fractal 1/f components using irregular resampling (IRASA). Window length was 5 s, 1 s sliding steps and modulating frequencies were estimated up to 6 Hz.

*Slow oscillations (SO):* Event detection was performed for every channel separately based on previously established algorithms^7,10^. In brief, we first filtered the continuous signal between 0.16 and 1.25 Hz (eegfilt.m) and detected all the zero crossings. Then events were selected based on time (0.8–2 s duration) and amplitude (75% percentile) criteria. Finally, we extracted 5-s-long segments (±2.5 s centered on the trough) from the raw signal and discarded all events that occurred during an IED.

*Sleep spindles:* We filtered the signal between 12 and 16 Hz (eegfilt.m) and extracted the analytical amplitude after applying a Hilbert transform (Fig. 1b). We smoothed the amplitude with a 200-ms moving average. Then the amplitude was thresholded at the 75% percentile (amplitude criterion) and only events that exceeded the threshold for 0.5–3 s (time criterion) were accepted. Events were defined as sleep spindle peak-locked 5-s-long epochs (±2.5 s centered on the spindle peak) and events that occurred during an IED were discarded.

*SO-Spindle co-occurrence (coupled vs. uncoupled events):* For all spindles, we quantified if a separate SO was detected independently in the same time interval (spindle peak ± 2.5 s) and spindles were classified into coupled or uncoupled events based on the simultaneous detection.

*Event-free, random intervals:* For control analyses, we also extracted random, non-overlapping 5-s-long intervals during NREM sleep, which did neither exhibit any SO and spindle events nor any artifactual IED activity. Onset samples were solely determined by the minimally required distance to all other events (2.5 s away from the previous SO trough or spindle peak).

*Ripples:* We utilized automatic IED detection algorithms to detect prominent discharges, however, this approach does not capture below-threshold epileptiform or artifactual activity in the HFB. Therefore, we imposed additional measures to isolate true ripples from artifactual ripples, which can easily be mistaken when data is analyzed in the frequency domain or after applying a band-pass filter (Fig. 4b). We first narrowed down our search window for ripples, to epochs during cortical spindle events (±1 s) during NREM sleep when no simultaneous IED had been detected. We time-locked the HFB traces relative to those spindle events and determined the strongest HFB peak during any given, artifact-free spindle event. Then we extracted the number of peaks in the raw signal in a 40-ms window around this peak. If equal or more than three peaks were detected, the event was classified as a true ripple, while events that only exhibited one or two peaks were classified as artifactual. Ripples were peak locked (Supplementary Fig. 4c). Note that for group averages (Fig. 4a, b), we followed the convention that ripples were nested in the upwards swing of the sharp wave and hence, we adjusted ripple polarity, which was less informative given the bipolar referencing scheme (Supplementary Fig. 4c). Resulting ripples were then visually inspected and we carefully assessed their spatiotemporal profile relative to other oscillatory events, such as slow oscillations and spindles (Fig. 4d–g).

*State-based connectivity analyses:* We calculated metrics for amplitude- as well as phase-based connectivity. Connectivity between the EEG and the MTL (Fig. 5a, b) was calculated for 25 logarithmically spaced bins between 0.5 and 32 Hz (±center frequency/4; eegfilt.m) in 30 s non-overlapping bins after band-pass filtering and applying a Hilbert transformation. Filtering was performed on continuous data to minimize filter edge artifacts. To assess envelope-based couplings, we calculated power correlations^36^. Even though the data was locally re-referenced (MTL: bipolar; EEG: common scalp EEG), we considered artifactual zero-lag interactions and removed them by orthogonalizing signals in the time domain prior to correlation analysis. The signals X* and Y* were derived by orthogonalizing the complex-valued signals *Y* to *X* and vice versa (Eq. (1)).

1 $$Y^ \ast = {\mathrm{imag}}\left( {Y\left( {t,f} \right) \times \frac{{{\mathrm{conj}}\left( {X\left( {t,f} \right)} \right)}}{{\left| {\left( {X\left( {t,f} \right)} \right.} \right|}}} \right)$$

Then orthogonalized correlations were calculated after extracting the squared analytic amplitude of the complex-valued signals and applying a log_10_ transform and averaged across both possible orthogonalized correlations (Eq. (2)).

2 $${\mathrm{rho}}_{\mathrm{{ortho}}} = \frac{{{\mathrm{corr}}\left( {X^ \ast ,Y} \right) + {\mathrm{corr}}\left( {Y^ \ast ,X} \right)}}{2}$$

Likewise, we removed zero-phase lag contributions from the phase-locking value (PLV) by only considering the magnitude of the imaginary part (iPLV; Eq. (3))^35^.

3 $${\mathrm{iPLV}} = \left| {{\mathrm{imag}}\left( {\frac{1}{N}\left( {{\mathrm{exp}}^{i\left( {\Phi _{{\mathrm{Signal}}\,1} - \Phi _{{\mathrm{Signal}}\,2}} \right)}} \right)} \right)} \right|$$

Connectivity between intracranial PFC and MTL electrodes was performed accordingly, however, here we obtained connectivity estimates for 34 logarithmically spaced bins between 0.5 and 152 Hz (Supplementary Fig. 5).

*Event-based connectivity analysis:* In order to resolve how the precise SO-spindle coupling phase indexes the PFC-MTL connectivity, we calculated the iPLV between the scalp EEG and all available MTL electrodes (Fig. 6). Data were epoched (±2.5 s) relative to all IED-free spindles in NREM sleep and spindles were grouped into 16 linearly spaced non-overlapping bins between –pi and pi. Given that different bins exhibited different event numbers, we repeated this procedure 50 times. On every iteration, we chose *n* trials, where *n* corresponds to 0.8x the smallest number in any given bin to obtain a bootstrapped distribution even from the smallest bin. Every segment was then transformed into the frequency domain by means of a fast Fourier transform after applying a Hanning window and the cross-spectrum (CSD) was extracted for all frequencies between 1 and 32 Hz (1 Hz steps; ft_freqanalysis.m) and the iPLV was derived from the CSD. The resulting iPLV spectrum was mean-normalized. To facilitate between subject comparisons and to account for the fact of varying preferred coupling directions, we aligned the average iPLV in the spindle range (12–16 Hz) to phase bin 9 (0–22.5°). This phase bin was excluded from subsequent testing.

*State-based directionality analysis:* Within frequency directionality was tested by means of the phase-slope index (PSI)^27^, which was calculated for non-overlapping 30 s segments based on the Fourier coefficients (Fig. 5c). Given the different number of wake and NREM epochs, we subsampled 0.8x the smallest number of events (50 repetitions) and averaged the resulting directionality spectra. While power differences potentially affect PSI estimates, they however do not affect directionality estimates, hence, values above zero indicate PFC to MTL directional influences, while values below zero signals the opposite directionality. We also compared it to a surrogate distribution as described below, which allowed us to only study NREM-specific effects and account for power differences between wake and NREM states.

*Event-based directionality analysis:* We repeated the analysis, but this time centered on the spindle events (Fig. 6d). Given that no spindles were present in the wake state, we compared the estimates relative to a surrogate distribution, where the PSI for event *N* (EEG channel) was calculated relative to event *N* + 1 (MTL channel)^7^. The last event was calculated relative to the first event.

*EEG SO-Spindle Cross-frequency coupling (CFC):* We first filtered the spindle peak-locked data (Fig. 1b, c) into the SO component (0.1–1.25 Hz) and extracted the instantaneous phase angle after applying a Hilbert transform and extracted the phase angle that coincided with a spindle peak. The mean circular direction (circ_mean.m) and resultant vector length (circ_r.m) across all artifact-free NREM events were determined using the CircStat toolbox^10,64^.

*EEG-MTL SO-Spindle to HFB coupling:* To analyze the triple coupling between these oscillations, we expressed the normalized HFB amplitude as a function of the precise SO-spindle coupling phase (Fig. 3d). First, we determined the SO coupling phase for every spindle event. We binned the SO-spindle coupling into 24 non-overlapping, 15° wide bins. Then, we time-locked the HFB activity relative to the detected cortical spindle events and extracted the average power per bin during the spindle peak. The resulting trace was smoothed with a 5-point boxcar window. To further quantify this relationship, we calculated a circular-linear correlation (circ_corrcl.m) between the phase vector (24 bins) and the resulting HFB estimates and z-scored the correlation value relative to a surrogate distribution (1000 repetitions) where the precise relationship between coupling phase and amplitude was abolished. The preferred coupling phase was determined as the phase bin that exhibited the highest HFB power.

We obtained a time course of this coupling interaction by repeating this process at every time point around the spindle event (Fig. 1h, i). We determined the optimal coupling phase for every subject and every time point and tested the phase consistency across subjects using a cluster-corrected Rayleigh test (circ_rtest.m; see below).

*MTL-EEG Ripple to SO and Spindles:* While we detected ripple events during spindle events (±1 s), this approach did not make any predictions about the exact temporal relationship between the phase of spindle event and the detected ripple peak. Likewise, it did not introduce a bias towards a specific SO phase, given that more than a whole SO cycle was captured within this time window. Therefore, we performed event-locked cross-frequency analyses after band-pass filtering the continuous scalp EEG data into either the SO band (0.1–1.25 Hz) or the spindle band (12–16 Hz) and applying a Hilbert transform to extract the analytic phase. Then we extracted all phase angles that coincided with a ripple peak and quantified the degree of non-uniformity using the CircStat toolbox. We furthermore counted the number of detected ripple events relative to the SO (−2 to 2 s around the SO trough; 100 bins). To minimize between-subject variability, the resulting distributions were mean-normalized and smoothed with a 10-point boxcar window. Then we tested when the ripple count was significantly above the mean across subjects and tested if more ripples occurred prior or after the down-state (400 ms window centered on the cortical up-state before and after the downstate (Fig. 4d, e).

*State-based cross-frequency coupling* (Fig. 2f, g): In addition to the event-locked analyses, we also screened a wider range of possible phase-amplitude pairs. Therefore, we filtered the continuous data into distinct frequency bands and then calculated the Modulation Index^31^ for non-overlapping 30 s segments. We extracted the phase from the EEG signal after band-pass filtering the data into 25 logarithmically spaced bins between 0.5 and 32 Hz (±¼ center frequency; eegfilt.m). Amplitude series were extracted for all available MTL channels in 35 logarithmically spaced bins between 8 and 152 Hz. The band-pass was adjusted to include the side peaks of the low-frequency component^72^. Hence, the window at a given frequency was always defined as twice the low center frequency +2 Hz. The modulation index was then calculated after binning the low-frequency phase into 18 non-overlapping 20° wide bins^31^. For every bin, the mean amplitude was calculated and normalized before computing the normalized Kullback-Leibler divergence, which quantifies the deviation from a uniform distribution.

*Within region Cross-frequency directionality (CFD):* We assessed whether low frequencies components drive sleep spindle activity during SWS (Fig. 1d) or vice versa by calculating the cross-frequency phase-slope index^28,73^ between the normalized signal and the signal filtered in the sleep spindle range (12–16 Hz). We considered all SO frequencies <1.25 Hz after applying a Hanning window and extracting the complex Fourier coefficients. Significant values above zero indicate that SO drive sleep spindle activity, while negative values indicate that sleep spindles drive SO. Values around zero indicate no directional coupling.

*Across regions:* We further utilized directional CFC analyses^34,74^. Here directionality is commonly assumed if PAC (*Phase*_1_, *Amplitude*_2_) is larger than PAC (*Phase*_2_, *Amplitude*_1_) where signals 1 and 2 correspond to two spatially distinct regions. Here we tested if spindle-HFB coupling is stronger from PFC to MTL or vice versa (Fig. 2j).

*Mutual information and transfer entropy:* We tested if information transfer is enhanced between the MTL and PFC after a ripple by calculating time-resolved mutual information (MI; Fig. 7)^37^. We performed the analysis between the MTL channel that exhibited the highest percentage of physiologic ripples and one scalp electrode (typically Fz unless unavailable) or all available PFC electrodes. Data were epoched relative to the ripple and MI was calculated between −1 and +2.25 s in steps of 50 ms. Data was binned into 8 bins (uniform bin count; Fig. 7) within a 400-ms window centered on the current bin. Mutual information (Eq. (4)) between the two signals *X* and *Y* was defined as

4 $${\mathrm{MI}}\left( {X;Y} \right) = \mathop {\sum }\limits_{x \in X} \mathop {\sum }\limits_{y \in Y} p\left( {x,y} \right) \times {\mathrm{log}}_2\left( {\frac{{p\left( {x,y} \right)}}{{p\left( x \right) \times p\left( y \right)}}} \right)$$

where *p*(*x*, *y*) depicts the joint probability function and *p*(*x*) and *p*(*y*) indicate the class probabilities. Probabilities were normalized by their sum. MI traces in the time domain were mean-normalized relative to the individual baseline (−1 to 0 s). In order to test directional influences and given the uncertainty about the precise time lag between MTL and PFC, we calculated the MI during the ripple peak in either the MTL or the PFC and all previous and subsequent time bins in the other region (Fig. 7b, c). Likewise, we compared MI evolution after a physiologic and artifactual ripple relative to their individual baseline values (Fig. 7g, h). To assess the relationship of spindle peaks and MI peaks, we counted the number of spindle events in 120 evenly spaced bins (±3 s), which were mean normalized and smoothed with a 3-point boxcar function (Fig. 7e).

Spectrally resolved MI (Fig. 7b, c) was calculated after band-pass filtering the data into 25 logarithmically spaced bins between 1 and 64 Hz (±¼ center frequency) and extracting the instantaneous amplitude using a Hilbert transform. Spectrally resolved MI was normalized by means of a *z*-score per frequency band to discount the 1/f drop-off.

Phase transfer entropy (PTE; Eq. (5)) was calculated as an information-theoretical directional interaction metric^75,76^ according to the following formula.

5 $$PTE\left( {X,Y} \right) = \mathop {\sum }\nolimits^ p\left( {Y_\delta } \right)p\left( Y \right)p\left( X \right)\,{\mathrm{log}}_2\frac{{p\left( {Y_\delta \left| Y \right.,X} \right)}}{{p\left( {Y_\delta \left| Y \right.} \right)}}$$

Binning was again performed in eight bins after band-pass filtering, applying a Hilbert transform and extracting the instantaneous phase. The analysis was focused on a 1-s segments centered on every time point (±0.5 s; from −1 and +2.25 s in steps of 50 ms) and calculated between the prefrontal EEG and the MTL. The prediction delay δ was adjusted per frequency depending on phase changes sign across time^76^. To normalize the spectrum, we divided the PFC-to-MTL estimates by the MTL-to-PFC estimates; hence, values above 1 reflect information flow from PFC to the MTL.

*Relationship of MI and Spindle activity* (Fig. 7e, f)*:* For every physiologic ripple event, we extracted the subsequent time course of directional MTL-to-EEG information flow (Fig. 7c). Then we determined the largest MI peak for this given trial after the ripple event. Next we detected where the closest spindle peaked relative to this MI peak. Then we binned the obtained time stamps into 100 evenly spaced bins (range: ±2 s around MI peak). To reduce inter-subject variability, we normalized the resulting histogram by its mean prior to averaging. We tested where the observed distribution was significantly larger than one by means of a cluster-based permutation test. MI was also calculated on longer timescales (Fig. 7f) using the same settings, smoothed with a 20 point moving average and then *z*-scored for display purposes. The average phase difference between MI and spindle power (inset Fig. 7f) was calculated after band-pass filtering between 0.3 and 0.5 Hz and applying a Hilbert transform to extract the analytic phase.

### Statistical analysis

We used fixed effects analyses with the only exception of Supplementary Fig. 3 where we investigated the spatial extend of HFB modulations and given that different subjects contributed different numbers of electrodes, we utilized a random effects analysis. Unless stated otherwise, we used cluster-based permutation tests^77^ to correct for multiple comparisons as implemented in FieldTrip (Monte Carlo method; 1000 iterations; maxsum criterion). Clusters were formed in the time, frequency or time-frequency domain (e.g., Figs. 5a–c, 6d, and 7a, b, d) by thresholding two-tailed dependent *t*-tests at *p* < 0.05 unless stated otherwise. A permutation distribution was then created by randomly shuffling labels. The permutation *p*-value was obtained by comparing the cluster statistic to the random permutation distribution. The clusters were considered significant at *p* < 0.05.

Circular statistics were calculated using the CircStat toolbox. Circular non-uniformity was assessed with Rayleigh tests at *p* < 0.05 or the V-test if a mean direction was hypothesized based on previous evidence. Cluster testing for circular data (Fig. 3h) was based on Rayleigh tests (cluster threshold *p* < 0.05; maxsize criterion) and considered significant at *p* < 0.05. Effect sizes were quantified by means of Cohen’s d, the correlation coefficient rho or *η*^2^ in case of repeated measures ANOVAs. For circular statistics, we report the mean resultant vector length (mvl) as the effect size metric. To obtain effect sizes for cluster tests, we calculated the effect size separately for all data points and averaged across all data points in the cluster. Repeated-measures ANOVAs were Greenhouse-Geisser corrected. In case of unequal variances (Fig. 3j), we utilized the non-parametric Wilcoxon rank sum test. If test statistics were calculated on the individual subject level, we inferred significance at the group level based on a binomial distribution.

### Reporting Summary

Further information on research design is available in the Nature Research Reporting Summary linked to this article.

## Supplementary information

Supplementary Information

Reporting Summary

## Data Availability

The data sets generated and analyzed here are available from Dr. Jack Lin (linjj@uci.edu) on reasonable request.

## Code availability

All the computer code used to implement the experiments and to collect and analyze data is available from the corresponding author on reasonable request.
