# Supplementary material for: Bidirectional prefrontal-hippocampal dynamics organize information transfer during sleep in humans
Source: Nat Commun. 2019 Aug 8;10:3572. doi: 10.1038/s41467-019-11444-x (PMC6687745; doi:10.1038/s41467-019-11444-x)
Supplement: Supplementary file 1 — Supplementary Information [file 41467_2019_11444_MOESM1_ESM.pdf]

SUPPLEMENTARY INFORMATION

Supplementary Table 1

Subject	Recording Duration	Total Sleep Time [h]	WASO [min]	N1 [min]	N2 [min]	SWS [min]	REM [min]
1	21:28 - 8:28	6.81	110	40	263	40	66
2	20:30 - 8:30	4.04	496	31	166	35	11
3	20:01 - 8:01	7.68	295	55	267	40	99
4	20:00 - 8:00	7.32	326	33	315	18	74
5	20:01 - 8:01	8.18	113	42	147	221	81
6	22:28 - 7:02	4.90	223	14	192	53	36
7	20:44 - 7:44	9.73	119	50	327	89	119
8	20:04 - 8:04	7.00	356	52	262	19	88
9	19:47 - 8:30	7.41	307	48	291	78	29
10	20:16 - 8:16	6.25	371	32	201	42	101
11	19:56 – 8:56	9.07	284	66	245	105	129
12	20:22 - 7:43	5.28	342	54	192	49	22
13	20:16 - 8:16	5.25	353	58	93	145	20
14	20:38 - 8:38	10.82	110	28	355	207	60
15	21:55 - 7:55	5.93	219	103	184	70	0
16	19:38 - 8:23	6.91	242	52	218	89	56
17	19:36 - 8:04	3.01	279	39	118	9	15
18	19:41 - 7:41	7.48	279	90	243	93	24
**Group**	**12.43±1.54h**	**6.84 ± 1.96**	**268 ± 106**	**49 ± 21**	**227 ± 72**	**78 ± 60**	**57 ± 40**

Standard sleep metrics

We aimed to include continuous ~12-13h recording blocks, which were roughly obtained between 8pm and 8am. However, in some subjects the recording started later or stopped earlier due to ongoing testing or clinical considerations. Sleep staging was the carried out on the continuous epochs (mean recording time 12.4h ± 1.5h; mean ± SD; range: 8.6h – 14h). Furthermore note that the rater flagged epochs of excessive noise as artifactual and hence, those epochs were excluded from staging. Note that wake-after-sleep-onset values (WASO) are inflated, given that the clinical routine often wakes patients around 6am and hence, the last two hours of the recording often have been spent awake.

Supplementary Table 2

Subject	CA1	CA3DG	Sub	ERC	PRC	PHG	WM
1	0	0	0	5	0	6	1
2	4	2	1	3	1	4	1
3	1	0	0	3	0	0	0
4	10	0	1	0	0	7	2
5	8	0	0	6	0	11	1
6	2	8	0	1	0	0	1
7	3	12	6	1	0	0	0
8	11	3	2	1	0	0	1
9	13	0	0	0	2	0	1
10	7	4	0	0	0	0	3
11	6	11	0	0	0	0	1
12	23	0	0	2	0	5	4
13	8	0	0	1	0	0	3
14	17	4	0	7	3	11	4
15	15	6	2	3	0	3	1
16	13	7	0	6	0	0	2
17	3	4	1	0	0	0	2
18	11	4	0	6	1	5	3
All	**155**	**65**	**13**	**45**	**7**	**52**	**31**

Recording locations for every subject. Given that we use bipolar referencing between two adjacent contacts, we included both locations in the table. Hence the total number of location (N = 368) reflects two times the number of electrodes (N = 184). Electrode count is divided by ROI: Hippocampal regions CA1, CA3 and dentate gyrus, Subiculum as well as the entorhinal cortex (ERC), perirhinal cortex (PRC) or white matter (WM).

Supplementary Figure 1

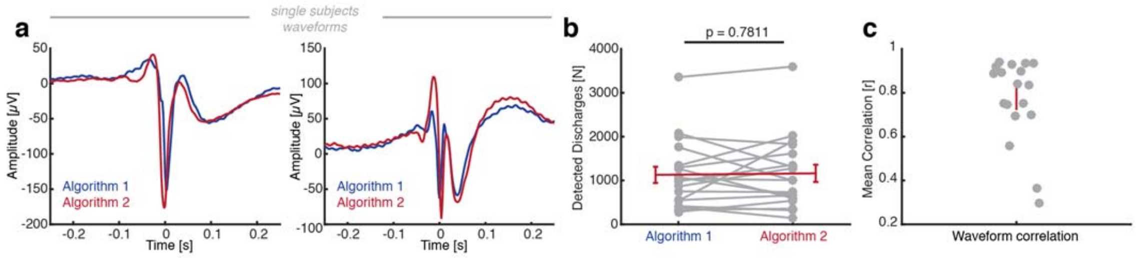

Interepileptic discharge (IED) characteristics

(a) Two single subject examples. We utilized two different algorithms to detect IED events[7,20], which yielded highly similar wave form shapes. (b) Both algorithms did not differ in the number of detected events (paired t-test: t_{17} = -0.28, p = 0.7811, d = 0.04; mean ± SEM in red). (c) The waveforms were strongly correlated (r = 0.77 ± 0.05, mean ± SEM in red; individual subjects in grey). Note that the seizure onset zone was outside of the MTL for subjects with mean correlation values of r < 0.5. To keep results comparable, we also removed MTL events that were labeled as epileptic for subjects who did not suffer from MTL epilepsy.

Supplementary Figure 2

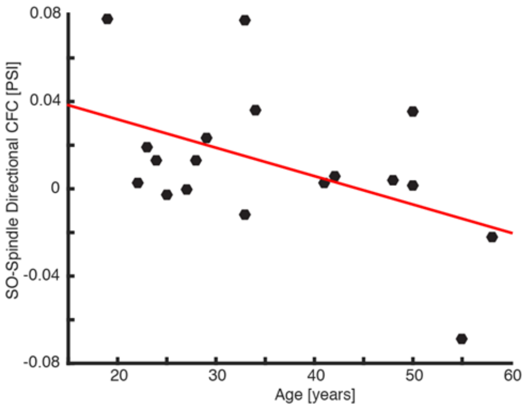

Age predicts directional SO-Spindle coupling

Linear correlation between age and the magnitude of directional CFC as measured by the PSI. We found a significant negative correlation between age and directional CFC ($r = -0.48$, $p = 0.0431$), thus, replicating our previously reported results[10].

Supplementary Figure 3

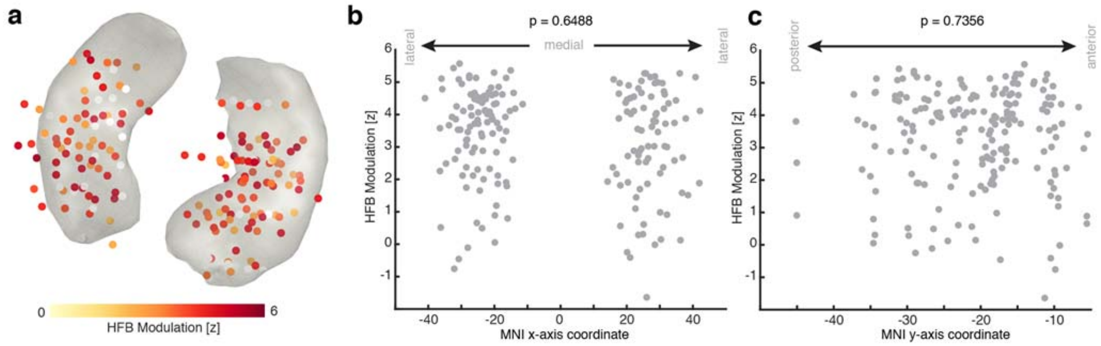

Distribution of HFB modulation in the hippocampus

(a) Topographical depiction on a standard MNI mesh. Color intensity reflects how strongly the SO-spindle coupling phase modulated the HFB amplitude. Values are masked at a z-score of 1.96 (white electrodes do not show a significant effect; all color-coded electrodes show a significant effect at uncorrected $p < 0.05$). (b) We tested whether the coupling varied between more medial and more lateral contacts. However, different subjects contributed a different number of electrodes to the analysis. Therefore, these observations were not independent. To account for dependence, we utilized a linear mixed-effects model to compare the HFB modulation as a function of the absolute value of the MNI coordinate to account for the symmetric distribution across hemispheres. Subjects were modeled as random effects. However, we did not find a systematic pattern (model estimate = -0.008, $t_{182} = -0.46$, $p = 0.6488$, 95%CI = [-0.04 0.03]). (c) We also tested whether the HFB modulation changed along the antero-posterior axis of the hippocampus, but again found no evidence for this consideration (model estimate = 0.004, $t_{182} = 0.34$, $p = 0.7356$ 95%CI = [-0.02 0.03]).

Supplementary Figure 4

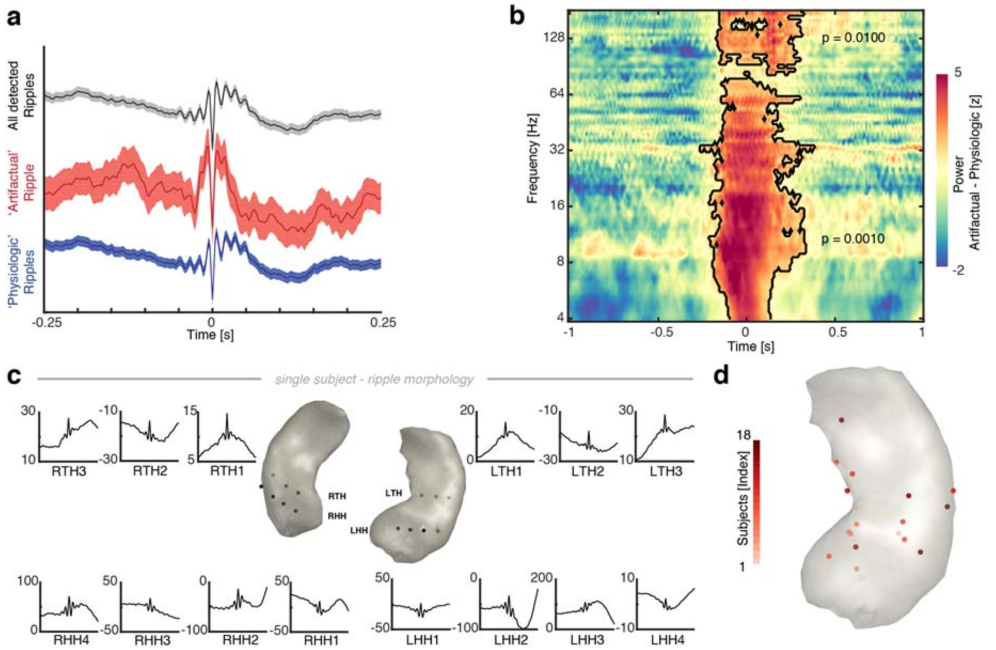

Detection of physiologic and pathologic ripple events

(a) Top: All detected ripple events in a single subject (mean ± SEM). Center: Ripple events that exhibited less than three distinct peaks in the raw signal during an increase in HFB activity during a spindle event. Bottom: Ripple events that exhibited multiple distinct peaks in the raw signal and met the criteria for a physiologic ripple. (b) Cluster-based permutation test for the multi-tapered time-frequency representation of physiologic and artifactual ripples. Artifactual detections reflected sharp transients in the signal, which exhibit high power across the whole spectrum, while true ripples only have power in a narrow high frequency band, suggesting the presence of a true oscillation. In accordance with this consideration, we found that artifactual ripples exhibited significantly more power in lower frequencies (< 64 Hz; $p = 0.0010$, $d = 1.15$) as well as increased power in high frequencies (> 128 Hz; $p = 0.0100$, $d = 0.76$), while no significant

differences were detected in the true ripple frequency band (~80-120 Hz). (c) Morphology of detected average ripple events across multiple contacts in a single subject who did not suffer from MTL epilepsy (seizure onset zone was in premotor cortical regions) to highlight the variability. (d) Schematic depiction of the selected ripple channels for all subjects projected onto a left hippocampal MNI mesh (we flipped the x-axis for N = 5 subjects, where the highest number of ripples was detected in the right hemisphere). All electrodes picked up activity from CA1, CA3/DG, Subiculum or the entorhinal cortex.

Supplementary Figure 5

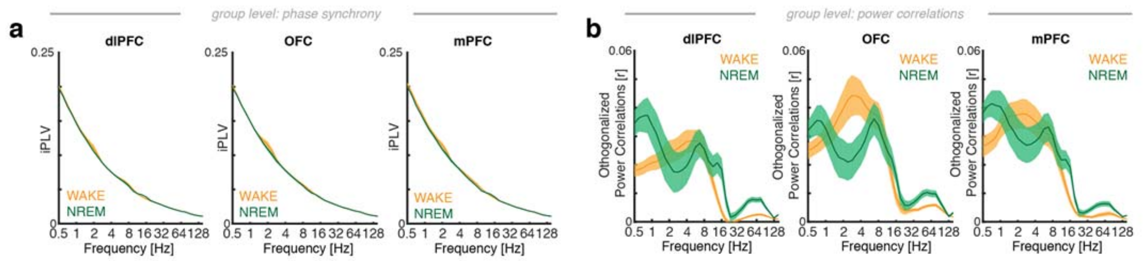

Interregional connectivity control analyses

(a) Phase coupling spectra for the three prefrontal ROIs, note that effects were generally small when averaged across the whole state (30s segments) given that oscillatory events during NREM sleep are short-lasted. (b) State-based amplitude-based coupling for the three different prefrontal ROIs (mean ± SEM). Note that the pattern mimicked the connectivity profiles as observed at scalp EEG level (**Fig. 5a/b**).

Supplementary Figure 6

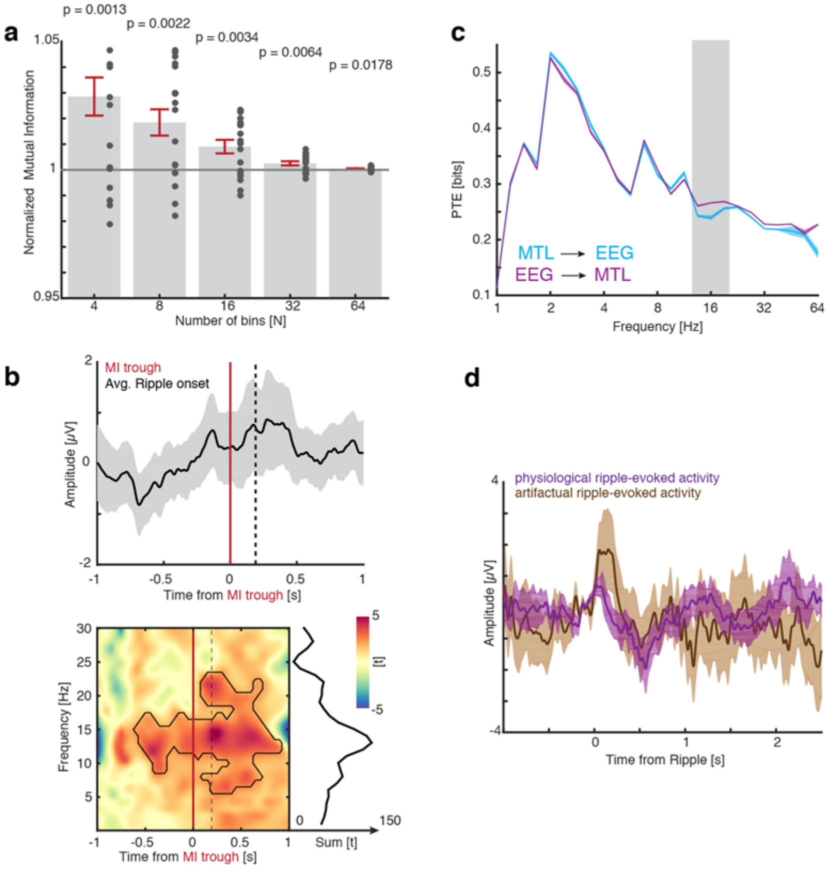

Information transfer between MTL and PFC

(a) To test the effect of bin size on mutual information estimates, we repeated the calculation based on a different number of bins and found that the effect could be reliably detected independent of the bin size. Note more bins decreased the absolute value as well as the variance across subjects. Hence, the results were qualitatively similar independent of the chosen bin size (mean ± SEM; grey dots depict individual subjects) in the significant time window (Fig. 7a, N = 8 bins) with comparable effect sizes (4 bins: $t_{17} = 3.86$, $p = 0.0013$, $d = 1.29$; 8 bins: $t_{17} = 3.61$, $p = 0.0022$, $d = 1.20$; 16 bins: $t_{17} = 3.41$, $p = 0.0034$, $d = 1.14$; 32 bins: $t_{17} = 3.11$, $p = 0.0064$, $d = 1.03$; 64 bins: $t_{17} = 2.62$, $p = 0.0178$, $d = 0.87$). (b) In **Fig.**

7b, we observed a non-significant trough in the MI trace just prior to the ripple. To investigate the electrophysiological underpinnings, we first identified the precise time point trough in every individual and then re-aligned the scalp EEG data to this time point (t = 0; dark red, black dashed line indicates average ripple onset). Upper panel: MI trough-locked potentials did not exhibit any prominent signatures in the time domain. Lower panel: However, in the frequency domain the MI trough is closely associated with spindle activity (cluster test: $p < 0.001$, d = 0.98). Given the time-frequency trade-off and the absence of clear time-locked spindle activity (upper panel), we speculate that this observation may reflect a prefrontal trigger signal, which is transmitted to the MTL to initiate the subsequent processing cascade. (c) Raw phase transfer entropy spectra for MTL to EEG (cyan) and EEG to MTL (purple) interactions during the ripple (± 0.5s). Note the prominent 1/f drop-off. Strong low frequency components do not exhibit a preferred directionality. The grey shaded area indicates the significant differences as reveled by a cluster-based permutation test. (d) The MI difference (**Fig. 7g/h**) could not be explained by differences in evoked activity following a physiologic or pathologic ripple (no clusters at $p < 0.05$).
