# Supplementary material for: Bidirectional prefrontal-hippocampal dynamics organize information transfer during sleep in humans
Source: Nat Commun. 2019 Aug 8;10:3572. doi: 10.1038/s41467-019-11444-x (PMC6687745; doi:10.1038/s41467-019-11444-x)
Supplement: Supplementary file 2 — Reporting Summary [file 41467_2019_11444_MOESM2_ESM.pdf]

nature research

Corresponding author(s): Randolph Helfrich

Last updated by author(s): 2019/07/07

Reporting Summary

Nature Research wishes to improve the reproducibility of the work that we publish. This form provides structure for consistency and transparency in reporting. For further information on Nature Research policies, see Authors & Referees and the Editorial Policy Checklist.

Statistics

For all statistical analyses, confirm that the following items are present in the figure legend, table legend, main text, or Methods section.

n/a	Confirmed	
☐	☒	The exact sample size (n) for each experimental group/condition, given as a discrete number and unit of measurement
☐	☒	A statement on whether measurements were taken from distinct samples or whether the same sample was measured repeatedly
☐	☒	The statistical test(s) used AND whether they are one- or two-sided<br>*Only common tests should be described solely by name; describe more complex techniques in the Methods section.*
☐	☒	A description of all covariates tested
☐	☒	A description of any assumptions or corrections, such as tests of normality and adjustment for multiple comparisons
☐	☒	A full description of the statistical parameters including central tendency (e.g. means) or other basic estimates (e.g. regression coefficient) AND variation (e.g. standard deviation) or associated estimates of uncertainty (e.g. confidence intervals)
☐	☒	For null hypothesis testing, the test statistic (e.g. F, t, r) with confidence intervals, effect sizes, degrees of freedom and P value noted<br>*Give P values as exact values whenever suitable.*
☒	☐	For Bayesian analysis, information on the choice of priors and Markov chain Monte Carlo settings
☒	☐	For hierarchical and complex designs, identification of the appropriate level for tests and full reporting of outcomes
☐	☒	Estimates of effect sizes (e.g. Cohen's d, Pearson's r), indicating how they were calculated

Our web collection on statistics for biologists contains articles on many of the points above.

Software and code

Policy information about availability of computer code

Data collection	Nihon Kohden recording software for clinical EEG data.
Data analysis	MatLab 2015a (MathWorks Inc.), using custom code as well as functions from the EEGLAB toolbox, the FieldTrip toolbox, the CircStat toolbox, Freesurfer and SPM12

For manuscripts utilizing custom algorithms or software that are central to the research but not yet described in published literature, software must be made available to editors/reviewers. We strongly encourage code deposition in a community repository (e.g. GitHub). See the Nature Research guidelines for submitting code & software for further information.

Data

Policy information about availability of data

All manuscripts must include a data availability statement. This statement should provide the following information, where applicable:

- Accession codes, unique identifiers, or web links for publicly available datasets
- A list of figures that have associated raw data
- A description of any restrictions on data availability

The data sets generated and analyzed here are available from Dr. Jack Lin (linjj@uci.edu) on reasonable request.

Field-specific reporting

Please select the one below that is the best fit for your research. If you are not sure, read the appropriate sections before making your selection.

☒ Life sciences ☐ Behavioural & social sciences ☐ Ecological, evolutionary & environmental sciences

October 2018

For a reference copy of the document with all sections, see nature.com/documents/nr-reporting-summary-flat.pdf

Life sciences study design

All studies must disclose on these points even when the disclosure is negative.

Sample size	No sample size calculation was performed. Participants were recruited continuously from UCI Medical Center depending on the electrode coverage. We included 18 subjects, which is 50% more than what comparable studies (e.g. Staresina et al. 2015 NatNeuro) reported.
Data exclusions	No data was excluded.
Replication	We performed both single subject as well as group level analyses. Single subject effects were replicated across multiple subjects and individual test statistics (i.e. z-scores) are provided.
Randomization	N/A
Blinding	N/A

Reporting for specific materials, systems and methods

We require information from authors about some types of materials, experimental systems and methods used in many studies. Here, indicate whether each material, system or method listed is relevant to your study. If you are not sure if a list item applies to your research, read the appropriate section before selecting a response.

Materials & experimental systems

n/a	Involved in the study
☒	☐ Antibodies
☒	☐ Eukaryotic cell lines
☒	☐ Palaeontology
☒	☐ Animals and other organisms
☐	☒ Human research participants
☒	☐ Clinical data

Methods

n/a	Involved in the study
☒	☐ ChIP-seq
☒	☐ Flow cytometry
☒	☐ MRI-based neuroimaging

Human research participants

Policy information about studies involving human research participants

Population characteristics	Human epilepsy patients who were implanted with intracranial depth electrodes for seizure onset localization.
Recruitment	Patients with intracranial EEG coverage in the MTL and PFC were included in the study on a continuous basis at UCI Medical Center.
Ethics oversight	Both UC Irvine (2014-1522) and UC Berkeley (2010-02-783) IRB approved the study.

Note that full information on the approval of the study protocol must also be provided in the manuscript.
